# Early-life viral infection generates pathological tissue-resident memory cells that contribute to asthma-like disease

**DOI:** 10.1172/jci.insight.198712

**Published:** 2026-03-10

**Authors:** Emma E. Brown, Jie Lan, Olivia B. Parks, Li Fan, Dequan Lou, Alysia McCray, Lisa Mathews, Alexander J. Wardropper, Anna Shull, Michelle L. Manni, Heth R. Turnquist, Kong Chen, Taylor Eddens

**Affiliations:** 1 University of Pittsburgh School of Medicine, Department of Pediatrics, Pittsburgh, Pennsylvania, USA.; 2 University of Pittsburgh Medical Scientist Training Program, Pittsburgh, Pennsylvania, USA.; 3 University of Pittsburgh School of Medicine, Department of Medicine, Pittsburgh, Pennsylvania, USA.; 4 University of Pittsburgh School of Medicine, Department of Immunology and Starzl Transplant Institute, Pittsburgh, Pennsylvania, USA.; 5 Washington and Jefferson College, Washington, Pennsylvania, USA.; 6 University of South Carolina, Columbia, South Carolina, USA.; 7 University of Pittsburgh School of Medicine, Department of Pharmacology and Chemical Biology, Pittsburgh, Pennsylvania, USA.; 8 Institute for Infection, Inflammation, and Immunity in Children (i4Kids), Pittsburgh, Pennsylvania, USA.

**Keywords:** Immunology, Infectious disease, Pulmonology, Asthma, T cells

## Abstract

Viral lower respiratory tract infections are common early in life and are associated with long-term development of asthma, a chronic condition defined by reversible airflow obstruction secondary to inflammation. Understanding the immunological mechanism connecting these two pathologies observed early in life becomes imperative to guide therapeutic measures. To investigate this connection, neonatal (days 4–6) or adult mice were infected with human metapneumovirus (HMPV) followed by a secondary HMPV infection 6 weeks later. Mice initially infected as neonates demonstrated increased mucus production, eosinophil recruitment, airway hyperresponsiveness, and Th2 T cell differentiation after rechallenge compared with adult mice rechallenged with HMPV. Neonatal HMPV infection led to formation of Th2 clonally expanded tissue-resident memory (TRM) T cells that were absent after adult HMPV. FTY720-mediated disruption of lymphocyte circulation demonstrated that TRMs contributed to pathology. Local depletion of lung CD4^+^ T cells and JAK2 inhibition mitigated pathology. These findings suggest TRMs uniquely generated after early-life viral infection can contribute to Th2-driven asthma pathology.

## Abstract 2

This study demonstrates that early life viral infection induces a pathologic memory population that contributes to Th2 inflammation consistent with asthma, representing a novel therapeutic target

## Graphical abstract

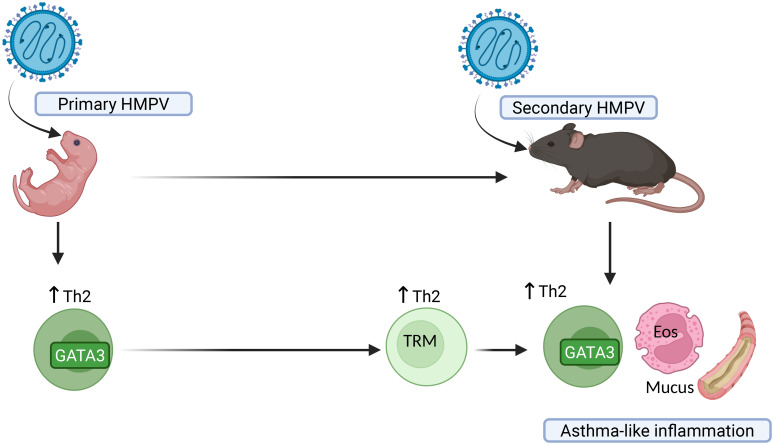

## Introduction

Asthma is the most common chronic condition in childhood and is defined by reversible airflow obstruction secondary to chronic inflammation (1). Exacerbations of asthma are most frequently caused by viral infections and can be life-threatening (2). Asthma is most commonly diagnosed within the first 4 years of life, highlighting the temporal overlap between the incidence of viral lower respiratory tract infections and physician-diagnosed asthma (3, 4). Asthma in this early life window has long been thought to be driven by type 2 inflammation, characterized by CD4^+^ Th2 cells, innate lymphoid cells, eosinophils, and mucus, consistent with the broader atopic march observed in this age (5–8). This may represent an oversimplification, however, as more recent studies have shown other inflammatory signatures in pediatric patients with asthma (9–12). Although the heterogeneity of asthma is apparent, this may in part be due to the multifactorial nature of asthma pathogenesis (e.g., genetics, in utero exposures, passive smoke exposure, air pollution).

Lower respiratory tract infections are the leading infectious cause of death in children under 5 years of age worldwide (13, 14). One of the most common and well-studied early-life exposures related to asthma development is respiratory viral infections. Viruses, including respiratory syncytial virus (RSV), human metapneumovirus (HMPV), influenza, and rhinovirus, are among the most prevalent pathogens causing early-life lower respiratory tract infections (15–19). Global estimates of yearly RSV and HMPV burden, as examples, are 33.2 and 14.2 million lower respiratory tract infections in children under 5 years old (15, 16). Children under the age of 1 and preterm infants are much more susceptible to poor outcomes with respiratory viruses (20, 21). Seropositivity studies of RSV and HMPV demonstrate near universal exposure to these viruses by age 3 (22–25). Despite this, reinfection occurs through the lifespan frequently, likely reflecting variation in viral strains and/or waning immunity (23, 26). HMPV infections have also been associated with triggering asthma exacerbations in children and adults, similar to other viral pathogens (27–31).

In addition to the acute morbidity and mortality from viral lower respiratory tract infection, these infections early in life have also been associated with long-term asthma development (32, 33). A lower respiratory tract infection within the first 3 years of life, regardless of the pathogen, was associated with an increased risk of asthma development by age 7 (34). Prospective studies on individual pathogens, including RSV, rhinovirus, and HMPV, have all demonstrated that early-life viral lower respiratory tract infections increase a child’s risk of developing recurrent wheeze or asthma by roughly 3- to10-fold (35–39). Although these studies often evaluated children with viral infections requiring medical attention, Rosas-Salazar et al. found that prospectively surveilled infants without evidence of RSV infection within the first year of life had a reduced risk of asthma development (40).

This clinical observation has been modeled previously using RSV. Early-life RSV infection in mice led to enhanced Th2 responses with RSV rechallenge in adulthood, contributing to asthma-like pathology (41, 42). A critical window early in life whereby viral infection confers an increased risk of asthma development may be in part due to immune differences with age. The immune responses to common stimuli in neonates and infants tend to favor antiinflammatory and tolerogenic responses compared with adult counterparts (43, 44). CD4^+^ T cells exemplify this concept; in infants, CD4^+^ T cells preferentially differentiate into Th2 and regulatory T cells rather than inflammatory antiviral Th1 cells (45–47). This process is tightly regulated, both by intrinsic epigenetic factors and extrinsic antiinflammatory, pro-Th2 crosstalk within the lung (48–53). Although the infant immune system has myriad differences compared with the adult immune system (3), the Th2 dominance established early in the lung may play a role in the persistent immune response observed in asthma.

Consistent with the prior literature, we have previously shown that HMPV infection in a neonatal HMPV model generates fewer Th1 cells and increased Th2 cells compared with adult mice infected with HMPV (53, 54). In the current study, we sought to assess whether this early-life Th2 response would persist and contribute to asthma after HMPV reinfection. Mice infected with HMPV as neonates mounted a Th2-skewed response in adulthood, recapitulating the clinical phenomenon of asthma development after early-life viral infection. This pathological response is mediated by a durable Th2 tissue-resident memory (TRM) population, presenting a possible therapeutic target for precise interventions to break the cycle of Th2-driven lung inflammation.

## Results

### Early-life HMPV infection leads to asthma-like pathology with reinfection.

**Figure 1:**
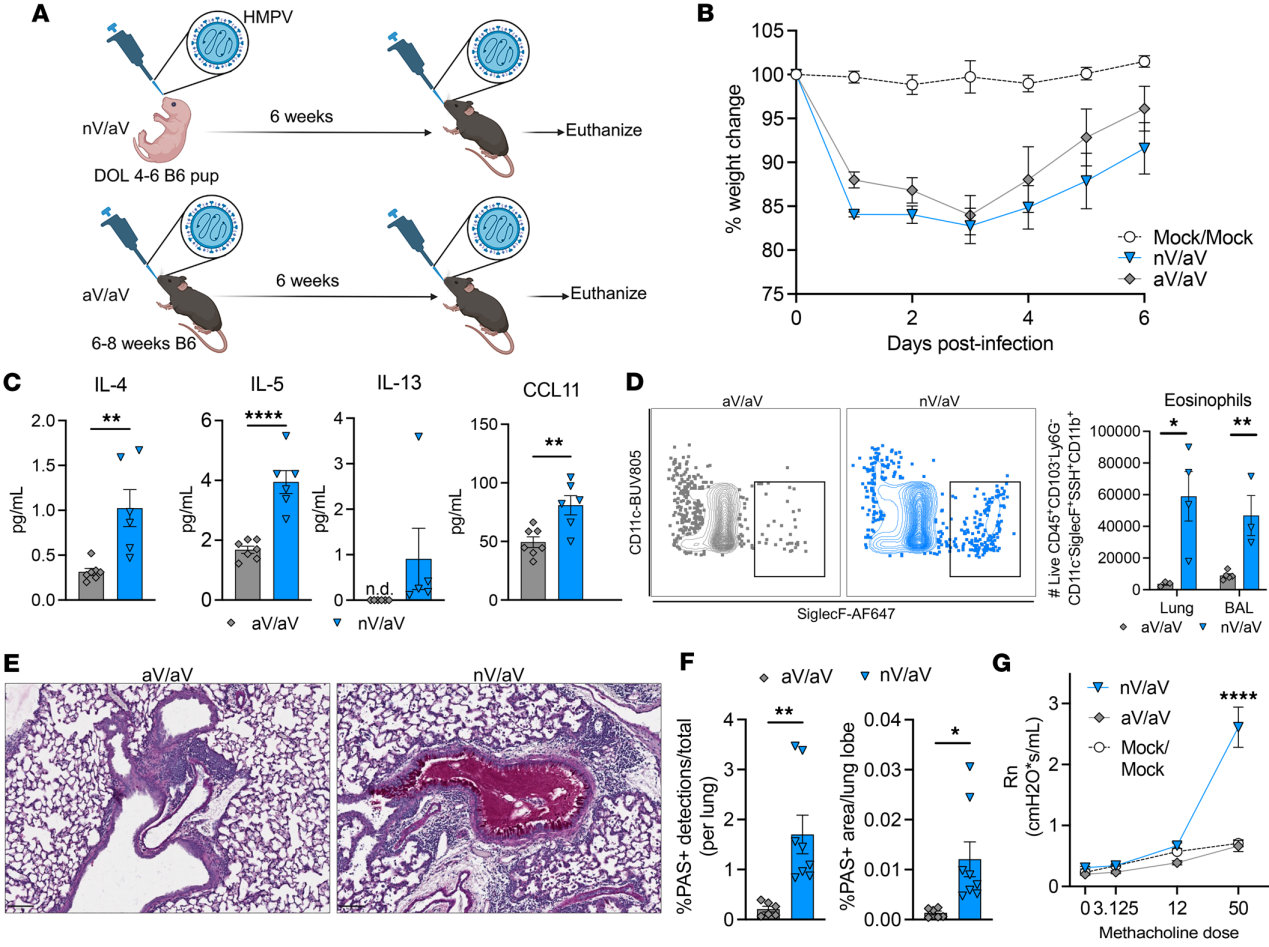
Early-life viral infection leads to skewed secondary response recapitulating asthma pathology. (**A**) Schematic of primary neonatal HMPV with secondary rechallenge in adulthood (nV/aV) or primary adult HMPV with rechallenge (aV/aV). Mice were euthanized at day 7 after rechallenge. Created with BioRender.com. (**B**) Weight loss was observed in both groups after rechallenge (*n* = 4–7/group). (**C**) IL-4, IL-5, IL-13, and CCL11 protein quantity from whole lung homogenate. **P < 0.01, ****P < 0.0005 by 2-tailed Student’s *t* test. (**D**) Representative flow plot of lung eosinophil identification (left) and enumeration (right) from lung homogenate and bronchoalveolar lavage (BAL) 7 days after rechallenge. *P < 0.05, **P < 0.01 by 2-tailed Student’s *t* test. (**E**) Periodic acid–Schiff (PAS) staining on FFPE lung tissue at day 7 after rechallenge showing mucus plugging in nV/aV mice. Scale bar: 100 μm (**F**) Quantification of PAS^+^ staining as percentage of total cell detections (left) and lung area (right). *P < 0.05, **P < 0.01 by 2-tailed Student’s *t* test. (**G**) FlexiVent analysis demonstrating airway hyperresponsiveness in nHMVP/aHMPV mice at 50 mg/mL methacholine administration. *n* = 2, 6, 5 for mock/mock, nV/aV, and aV/aV groups, respectively. ****P < 0.0005 by 2-way ANOVA with Dunnett multiple-comparison test.

To model the clinical phenomenon of early-life viral infection contributing to increased risk for asthma, we developed a HMPV reinfection model where the initial exposure occurred at different ages (Figure 1A). C57BL/6 mice were initially infected with HMPV as neonates (days 4–6) or adults (weeks 6–8), aged 6 weeks, and then reinfected with HMPV using a higher inoculum of the same strain as described previously (55). Mice initially infected with HMPV as neonates and reinfected as adults (nV/aV) showed similar weight loss as mice infected twice as adults (aV/aV, Figure 1B). nV/aV mice showed evidence of increased Th2 cytokine production in lung homogenate, with increased IL-4, IL-5, and CCL11 (eotaxin-1) (Figure 1C). Similarly, IL-13 was only detectable in nV/aV mice (Figure 1C). nV/aV had increased eosinophil (CD11c^–^SiglecF^+^CD11b^+^SSH^hi^) recruitment compared with aV/aV mice (Figure 1D). nV/aV had a robust mucus response on PAS staining, while aV/aV mice did not (Figure 1E). PAS detections were significantly increased in nV/aV mice compared with aV/aV mice (Figure 1F). Lastly, nV/aV had hyperresponsive airways upon methacholine challenge compared with aV/aV mice (Figure 1G). Collectively, these data demonstrate that early-life HMPV infection can alter the secondary response to HMPV in adulthood, resulting in asthma-like pathology.

This approach accounts for the timing of exposure and duration of immunological memory, but nV/aV mice were 6 weeks younger than aV/aV mice at the time of euthanasia. To test whether this age difference was a confounding variable, neonatal mice were infected with HMPV and aged 12 weeks, thereby matching the age of aV/aV 6 weeks after infection (Supplemental Figure 1A; supplemental material available online with this article; https://doi.org/10.1172/jci.insight.198712DS1). Further, nV mice were aged 20 weeks to extend the duration between rechallenges. Eosinophil recruitment was increased in the 12- and 20-week nV/aV models (Supplemental Figure 1B). Robust mucus production was observed in nV/aV mice in both the 12- and 20-week models (Supplemental Figure 1, C and D).

We next sought to assess whether live infection is required for induction of asthma-like pathology. To that end, we adapted our model to use UV-inactivated virus at either primary or secondary rechallenge. Compared with nV/aV controls, mice exposed to UV-inactivated virus both times (e.g., nUV/aUV) failed to upregulate mucus production (Supplemental Figure 2, A and B). nV/aUV and nUV/aV mice had similar PAS staining compared with nV/aV mice. nV/aV mice had a trend toward significantly increased eosinophil counts compared with nUV/aUV mice (Supplemental Figure 2C). From a CD4^+^ perspective, nV/aV, nV/aUV, and nUV/aV groups had similar number of GATA3^+^ CD4^+^ T cells in the lung (Supplemental Figure 2D). However, nV/aV mice had a significant expansion of Th1 Tbet^+^ cells compared with all other groups (Supplemental Figure 2E). nV/aV and nUV/aV mice were the only groups with detectable IL-4 and IL-13, while IL-5 showed no differences amongst groups (Supplemental Figure 2F). Collectively, these data would suggest Th2-driven pathology can occur if UV-inactivated virus is substituted for any single viral infection, but is less robust if only UV-inactivated exposures are used.

### Early-life HMPV infection results in a durable CD4^+^ Th2 TRM response.

**Figure 2:**
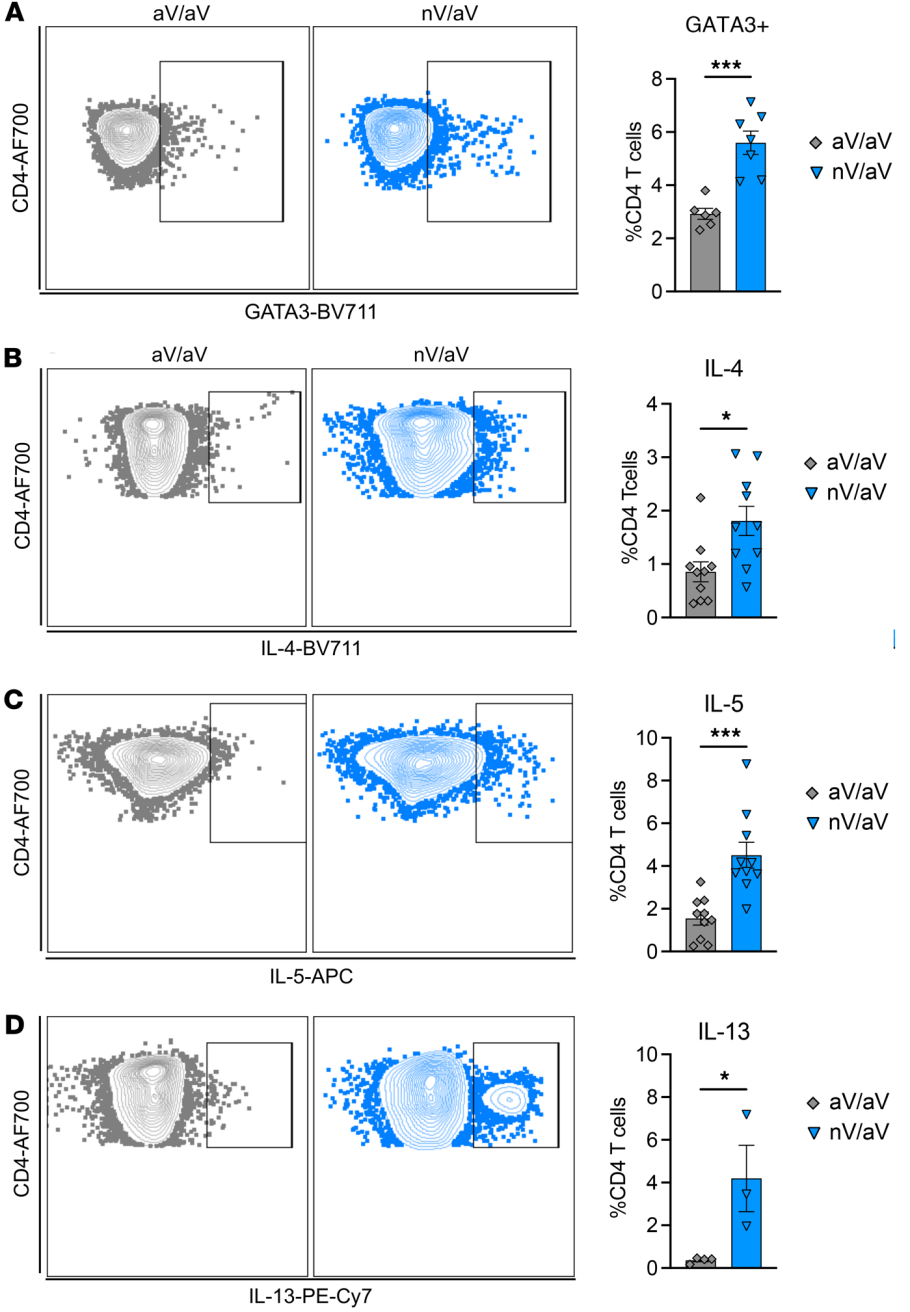
Early-life viral infection results in secondary response with increased CD4^+^ Th2 signature. (**A**) Representative flow plots and quantification of intracellular GATA3 staining in CD4^+^ T cells isolated from the lung at day 7 after rechallenge showing increased levels in nV/aV mice. (**B**–**D**) Intracellular staining of IL-4, IL-5, and IL-13 production after ex vivo HMPV peptide stimulation in lung CD4^+^ T cells 7 days after rechallenge. *P < 0.05, **P < 0.01, ***P < 0.005 by 2-tailed Student’s *t* test.

nV/aV mice had an increased proportion of CD4^+^ T cells that were GATA3^+^, the hallmark transcription factor for Th2 cells, compared with aV/aV mice (Figure 2A). Upon restimulation with an HMPV-specific peptide (N217), CD4^+^ T cells from nV/aV mice had increased IL-4, IL-5, and IL-13 production compared with aV/aV mice (Figure 2, B–D). Notably, there were no changes in Tbet^+^ or antigen-specific IFN-γ–producing CD4^+^ T cells between the nV/aV and aV/aV groups (Supplemental Figure 3, A and B). These data demonstrate that secondary T cell responses are different depending on the timing of initial exposure, suggesting that the skewed initial T cell responses in neonatal infection are durable (53).

**Figure 3:**
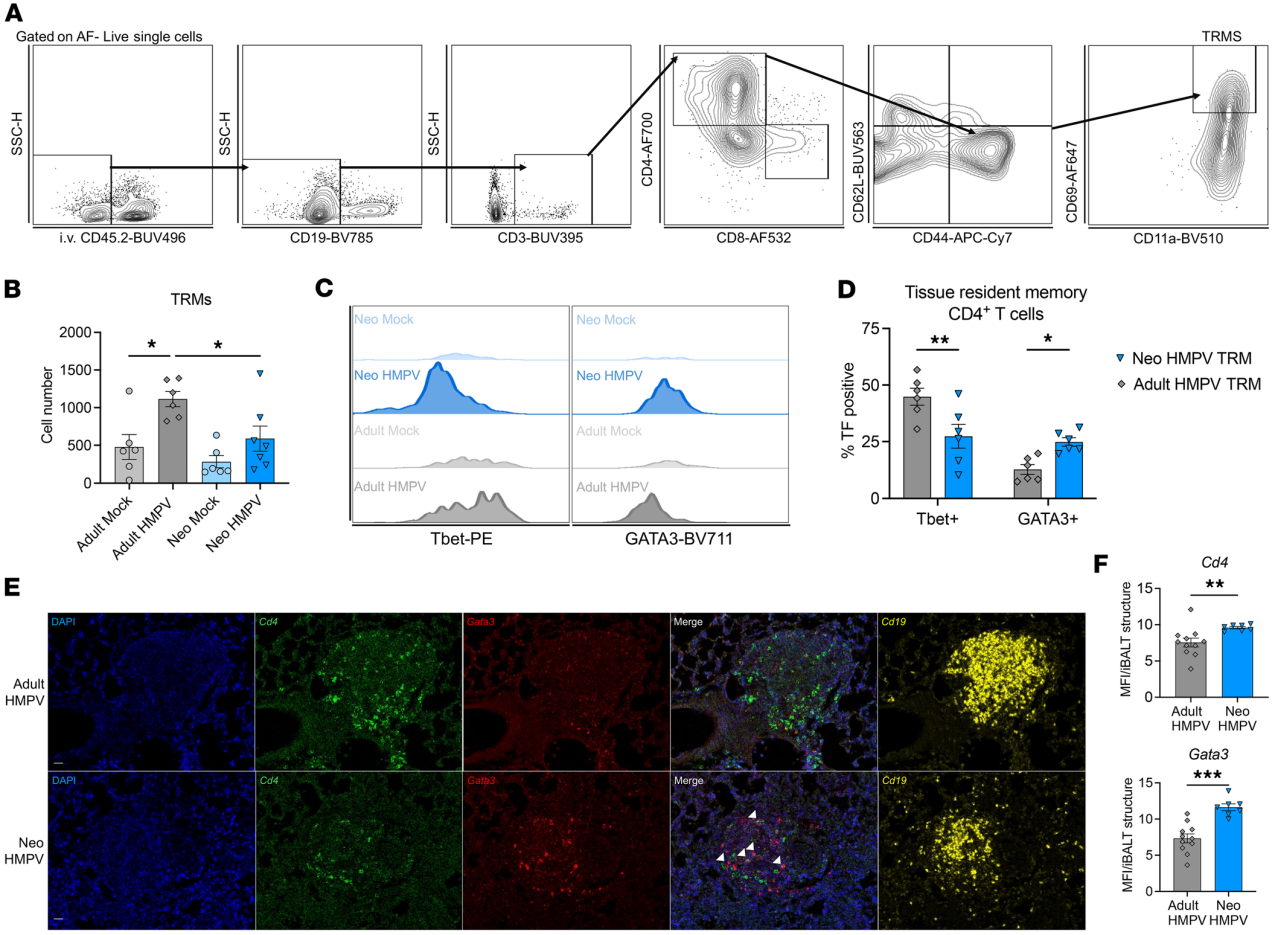
CD4^+^ tissue-resident memory cells are skewed toward a Th2 phenotype after early-life viral infection. (**A**) Gating strategy for isolation of tissue-resident memory (TRM) cells, including exclusion of intravascularly labeled cells (CD45.2^–^) and CD19^–^CD3^+^CD4^+^CD62L^–^CD44^+^CD11a^+^CD69^+^. (**B**) Quantification of TRMs 5 weeks after adult or neonatal HMPV infection (or mock infection). *P < 0.05 by 1-way ANOVA with Šidák’s multiple-comparison test. (**C**) Representative histograms of intracellular Tbet (left) and GATA3 (right) in TRMs isolated 5 weeks after neonatal or adult HMPV or mock infection. (**D**) Proportion of CD4^+^ T cells expressing Tbet or GATA3 in NeoTRM or AdultTRM. *P < 0.05, **P < 0.01 by 2-way ANOVA with Šidák’s multiple-comparison test. (**E**) Localization of GATA3^+^, CD4^+^, and CD19^+^ cells 5 weeks after adult or neonatal HMPV infection using RNAscope. (**F**) Quantification of CD4 and GATA3 MFI per individual iBALT structure. **P < 0.01, ***P < 0.005 by 2-tailed Student’s *t* test.

We hypothesized that TRM T cells were contributing to the Th2-driven pathology observed in the nV/aV model. Neonatal and adult mice were infected with HMPV, and TRMs were assessed 5 weeks after infection by intravascular labeling (56). After excluding labeled circulating cells, TRMs were identified by CD19^–^CD3^+^CD4^+^CD44^+^CD62L^–^CD11a^+^CD69^+^ populations (Figure 3A). Adult mice had a significant increase in TRM number after infection (Figure 3B). Adult HMPV-infected mice also had a significantly increased TRM number compared with neonatally infected mice (Figure 3B). TRMs from adult HMPV-infected mice had increased Tbet MFI and a greater proportion of Tbet^+^ cells compared with TRMs from mice infected with HMPV as neonates (Figure 3, C and D). In contrast, TRMs formed in the neonatal period had increased GATA3 MFI and significantly more GATA3^+^ cells compared with adult TRMs (Figure 3, C and D), consistent with Th2 functionality. After neonatal HMPV, GATA3^+^ T cells were significantly increased in number compared with the mock-infected animals, while no increase was observed after adult HMPV (Supplemental Figure 4A). In contrast, adult HMPV led to a significant increase in the number of Tbet^+^ cells (Supplemental Figure 4B). GATA3^+^CD4^+^ T cells formed after neonatal HMPV appeared to localize to organized tertiary lymphoid structures known as inducible bronchus-associated lymphoid tissue (iBALT, Figure 3E) (57, 58). GATA3^+^CD4^+^ T cells colocalized with CD19^+^ B cells near airways or vessels. iBALT structures were observed after adult HMPV infection, but GATA3^+^CD4^+^ cells were largely absent from these structures (Figure 3E). Further quantification demonstrated increased *Cd4* and *Gata3* signal in neonatal iBALT structures compared with adult iBALT structures (Figure 3F).

**Figure 4:**
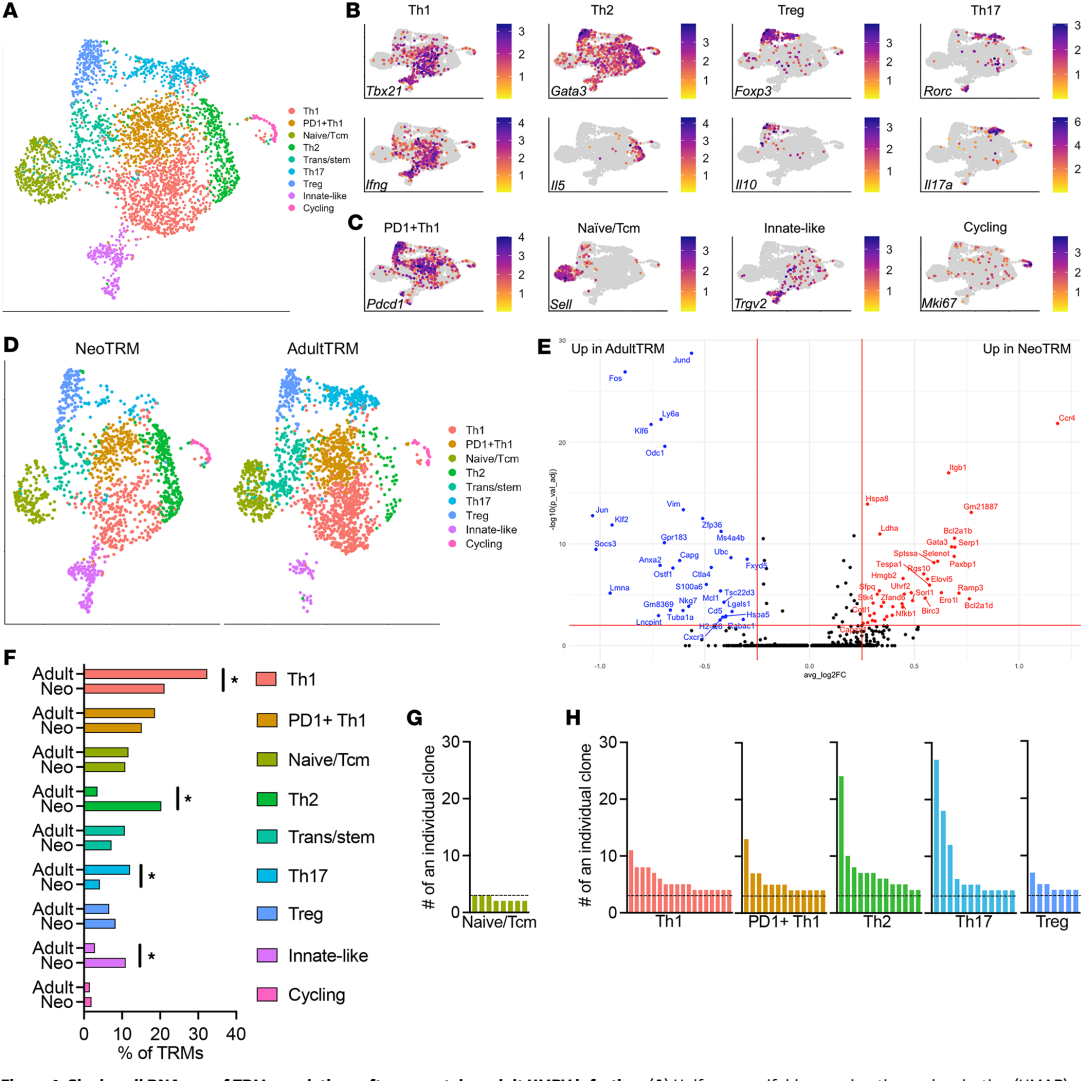
Single-cell RNA-seq of TRM populations after neonatal or adult HMPV infection. (**A**) Uniform manifold approximation and projection (UMAP) visualization of lung TRM populations isolated by flow-sorting 5 weeks after neonatal or adult HMPV infection demonstrating 9 clusters. (**B**) Expression of Th1, Th2, Treg, and Th17 transcription factors (e.g., *Tbx21*, *Gata3*, *Foxp3*, *Rorc*) and cytokines (*Ifng*, *Il5*, *Il10*, *Il17a*) demonstrating populations consistent with these established subsets. (**C**) Expression of markers corresponding to other cell states, including *Pdcd1* (PD-1^+^), *Sell* (CD62L, naive), *Trgv2* (innate-like T cell), and cycling (*Mki67*). Expression patterns in **B** and **C** and Supplemental Figure 2 were used to manually annotate the 9 clusters shown in **A**. (**D**) UMAP visualization of TRM clusters split by group (NeoTRM vs. AdultTRM). (**E**) Volcano plot of differentially expressed genes in pseudo-bulked NeoTRM versus AdultTRM cells. Genes displayed have *P*_adj_ < 0.01 and log_2_FC (≤0.25 or >0.25). (**F**) Proportion testing of individual clusters in NeoTRM versus AdultTRM. Significance defined as FDR < 0.05 and abs(log_2_FC > 0.58). (**G**) Clonotype analysis of TCR sequencing with each bar representing count of an individual TCR. Naive/Tcm cells had no more than 3 of the same TCR per cluster (dashed line). (**H**) Clonotype expansion in Th1, PD-1^+^ Th1, Th2, and Th17 clusters.

To further phenotype TRMs, 5 weeks after either neonatal or adult HMPV infection, TRM cells were flow-sorted on intravascularly-excluded CD4^+^ T cells and analyzed via single-cell RNA-seq. A total of 1,523 neonatal HMPV TRMs and 2,044 adult HMPV TRMs were sequenced (Supplemental Figure 5A). These cells had high expression of *Cd3e* and *Cd4* (Supplemental Figure 5B), with no detectable expression of *Cd8a* or *Cd19*. Markers of tissue residence, *Cxcr6*, *Itgal*, and *Cd69*, were also highly expressed in these cells (Supplemental Figure 5C). After identification of variable features, clustering analyses yielded 9 clusters, with some distinguishing markers corresponding to CD4^+^ T cell subsets or activation states (Figure 4A and Supplemental Figure 5D). These clusters could also be identified by transcription factors (*Tbx21* [gene for Tbet], *Gata3*, *Foxp3*, and *Rorc*) and cytokines (*Ifng*, *Il5*, *Il10*, and *Il17a*) associated with Th1, Th2, Treg, and Th17 subsets, respectively (Figure 4B). Chemokine receptors and surface markers associated with each subset were similarly identified (Supplemental Figure 5E). The Th1-like cells spanned 2 clusters, differentiated primarily by expression of *Pdcd1* (Figure 4C). Annotation of the remaining clusters included identification of naive cells (*Sell*), innate-like T cells (*Trgv2*), and cycling cells (*Mki67*) (Figure 4C). Lastly, a small cluster of transitional cells with stem-cell marker expression (e.g., *Tcf7*) resided in between naive and differentiated cells in the UMAP space (Figure 4A).

After cluster annotation, we next directly compared neonatal TRMs with adult TRMs (Figure 4D). Differential gene expression analyses demonstrated that neonatal TRMs had upregulation of *Ccr4* and *Gata3*, two hallmark Th2 genes (Figure 4E). The Th2 TRM cluster also expressed *Il4* and *Il13* (Supplemental Figure 5F). By comparison, adult TRMs had significant overrepresentation of *Cxcr3*, a Th1-associated chemokine receptor. There were notable differences in cluster abundance between neonatal and adult TRMs (Figure 4, D and F). Neonatal mice had a significantly increased proportion of Th2 TRMs and innate-like TRMs and a decreased proportion of Th17 and Th1 cells compared with adult mice (Figure 4F). Single-cell TCR sequencing was also performed to identify cells that were clonally expanded. As a comparator, the naive/Tcm cluster showed very little evidence of clonal dominance, with individual TCR sequences present in at most 3 specific cells (dashed line, Figure 4G). However, the Th1, PD1^+^ Th1, Th2, and Th17 clusters all showed evidence of expanded TCR sequences, indicative of clonally expanded cells (Figure 4H).

The finding of increased innate cells (Figure 4F) after neonatal HMPV raised the question of the role of type 2 innate lymphoid cells (ILC2s) in this model, as the IL-33/ST2 axis on ILC2s has been implicated in age-dependent RSV-driven pathology (59). ILC2s were detected after nV/aV challenge (Supplemental Figure 6A). To assess the role of this pathway in nV/aV asthma pathology, mice lacking *Il1rl1* (the gene encoding ST2) were infected with an expected diminution of the ST2^+^ ILC2 population (Supplemental Figure 6A). Eosinophil recruitment was similar between B6 and *Il1rl1*^–*/*–^ mice (Supplemental Figure 6B). GATA3^+^ CD4^+^ differentiation was slightly increased in *Il1rl1*^–*/*–^ mice, while Tbet^+^ T cell differentiation was significantly reduced (Supplemental Figure 6, C and D). Mucus production and pathological scoring were similar between B6 and *Il1rl1^–/–^* nV/aV mice (Supplemental Figure 6, E and F). These findings suggested nV/aV pathology did not require IL-33 signaling, prompting further evaluation of the tissue-resident T cells.

### TRM CD4^+^ T cell enhancement is sufficient for pathology.

**Figure 5:**
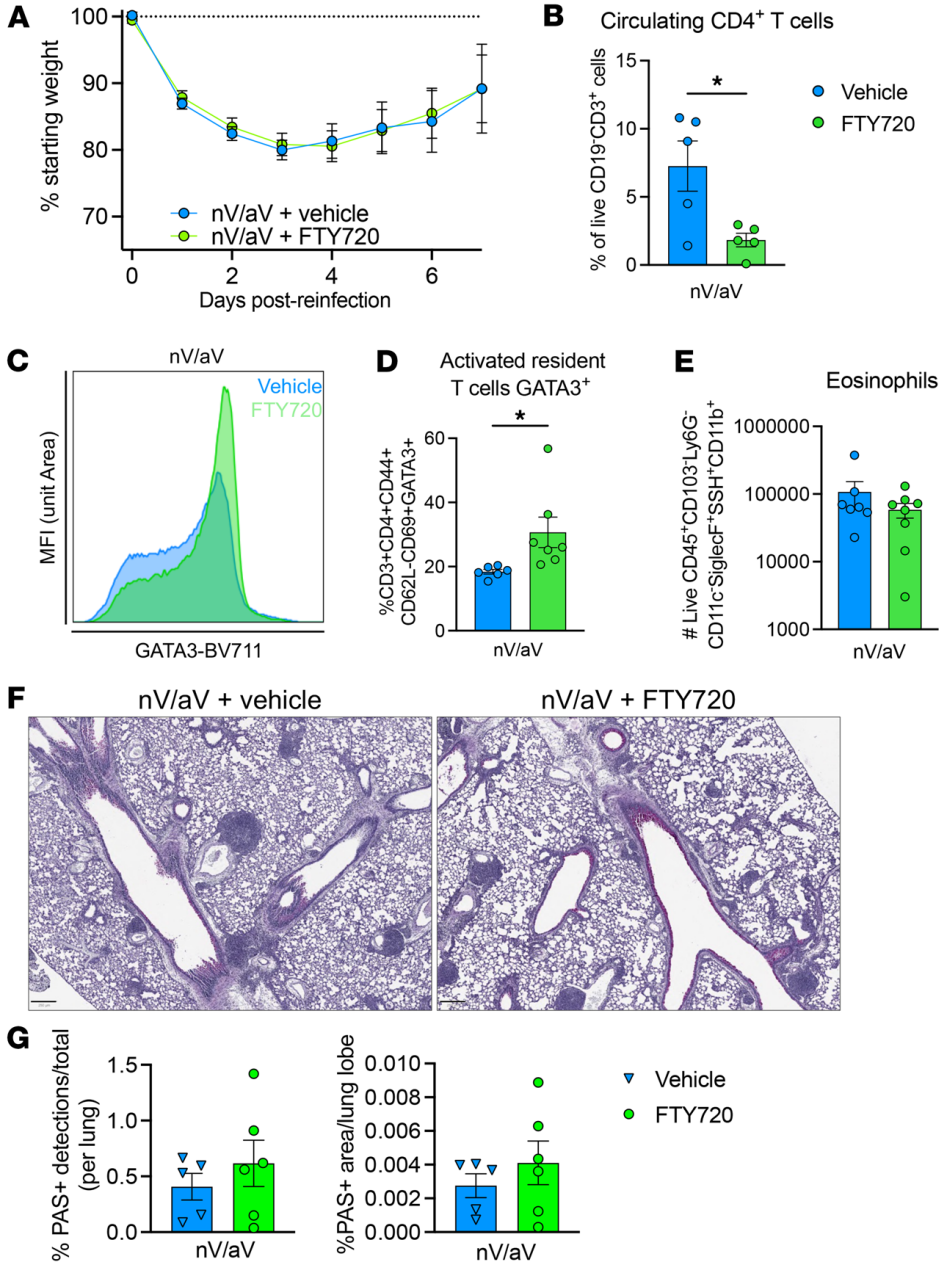
Lung-resident cells are sufficient to cause asthma-like pathology after early-life viral infection. (**A**) nV/aV mice were treated with vehicle or FTY720 (inhibitor of lymphocyte trafficking) for duration of experiment. Weight loss was similar between groups. (**B**) FTY720 significantly reduced blood CD4^+^ T cells. (**C** and **D**) Representative histogram and enumeration of activated GATA3^+^CD4^+^ T cells in the lung 7 days after rechallenge. (**E**) Enumeration of eosinophils in the lung 7 days after rechallenge. (**F** and **G**) PAS staining and quantification demonstrating mucus production was similar between groups. Scale bar: 250 μm. *P < 0.05 by 2-tailed Student’s *t* test.

To assess whether lung-resident cells are sufficient, nV/aV mice were treated with FTY720, a sphingosine-1-phosphate receptor modulator known to prevent T cell egress from the lymph node, thus limiting the response to cells already located in the tissue. FTY720 nV/aV mice had similar weight loss and recovery compared with vehicle-treated mice (Figure 5A). FTY720 treatment significantly reduced the circulating CD4^+^ T cell population (Figure 5B). In the lungs of FTY720-treated mice, a significant expansion of GATA3^+^CD4^+^ T cells was noted within the activated resident T cell population (Figure 5, C and D). Eosinophil recruitment was similar in vehicle- and FTY720-treated mice (Figure 5E). Mucus production was similar between vehicle and FTY720-treated mice (Figure 5, F and G). These data support the hypothesis that localized cells within the tissue are sufficient to induce the asthma pathology observed after early-life viral infection.

**Figure 6:**
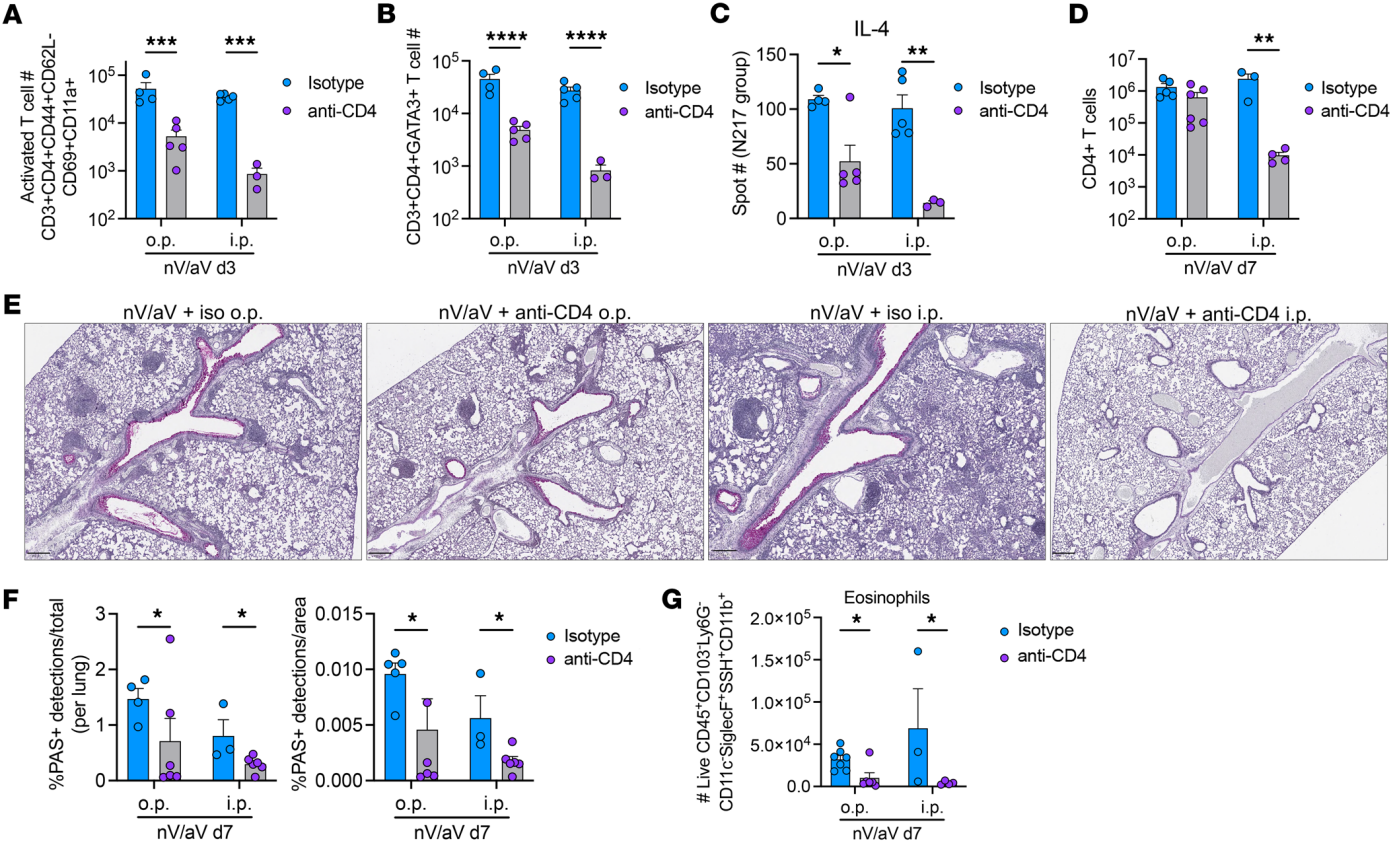
Local depletion of CD4^+^ T cells partially mitigates asthma pathology. Mice were treated with low-dose anti-CD4 antibody (100 μg; o.p.), high-dose anti-CD4 antibody (i.p.), or isotype control (o.p.) 1 day prior to HMPV reinfection. (**A**) Administration of o.p. and i.p. anti-CD4 antibody significantly reduced lung CD4^+^ T cells with an activated phenotype in the lung at day 3 after rechallenge. (**B**) Quantification of GATA3^+^CD4^+^ T cells after anti-CD4 treatment. (**C**) IL-4 production at day 3 after rechallenge was significantly reduced in mice treated with o.p. or i.p. anti-CD4. (**D**) Recovery of CD4^+^ T cells by day 7 after rechallenge was observed in o.p. but not i.p. anti-CD4–treated mice. (**E**) PAS staining demonstrating mucus production in isotype or antibody-treated groups at day 7 nV/aV. Scale bar: 250 μm. (**F**) Quantification of PAS^+^ staining as percentage of total cell detections (left) and lung area (right). (**G**) Eosinophil quantification after o.p. or i.p. treatment in mice at day 7 nv/aV. *P < 0.05, **P < 0.01, ***P < 0.005, ****P < 0.0005 by 2-way ANOVA with multiple comparisons.

To deplete local TRMs, nV/aV mice were treated with low-dose anti-CD4 antibody via oropharyngeal (o.p.) administration 1 day prior to reinfection or high-dose systemic (i.p.) anti-CD4 antibody as a positive control. Both approaches yielded significant reduction of lung CD4^+^ T cells at day 1 after treatment, but only o.p.-treated mice had recovery of CD4^+^ T cells to near-baseline levels by day 7 after infection (Supplemental Figure 7A). A similar effect was seen systemically, with o.p. treatment transiently reducing spleen and circulating CD4^+^ T cells (Supplemental Figure 7, B and C). At day 3 after reinfection, prior to recruitment of a secondary response, the total number of activated CD4^+^ T cells in the lungs was significantly reduced using both treatment strategies compared with mice receiving o.p. isotype control (Figure 6A). Similarly, GATA3^+^ CD4^+^ T cell number was reduced with o.p. anti-CD4 (Figure 6B). At this early time point, antigen-specific IL-4 response was modestly reduced in anti-CD4 o.p. nV/aV mice and completely abrogated in anti-CD4 i.p.-treated nV/aV mice (Figure 6C).

At day 7 after rechallenge, o.p. anti-CD4–treated mice had similar CD4^+^ T cell number compared with the isotype control, while i.p.-treated mice had significantly reduced CD4^+^ T cell number (Figure 6D), further demonstrating that o.p.-treated mice had a similar secondary response in magnitude. ILC2 number was unchanged with these interventions (Supplemental Figure 7D). nV/aV mice with o.p. and i.p. anti-CD4 treatment had significant reduction in mucus production compared with isotype nV/aV controls (Figure 6, E and F). Similarly, both o.p. and i.p. treatment led to a significant reduction in eosinophils (Figure 6G). The complete abrogation of CD4^+^ T cells demonstrated that these cells are necessary for nV/aV asthma pathology, and early treatment with anti-CD4 suggests a role for memory T cells in this process.

To further demonstrate that TRMs are sufficient, we next performed an adoptive transfer of either 5 × 10^5^ CD45.2^+^ NeoTRMs or AdultTRMs into naive CD45.1^+^ adult mice followed by challenge with high-dose HMPV (Supplemental Figure 8A). At 7 days after HMPV, 1%–2% of CD3^+^ T cells were CD45.2 transferred cells (Supplemental Figure 8B). Eosinophil recruitment was significantly increased in mice receiving NeoTRMs compared with mice receiving AdultTRMs (Supplemental Figure 8C). We also observed fascinating differences in T cell responses. Transferred NeoTRMs retained a significant increase in MFI of GATA3 and GATA3^+^ percentage in activated T cells in the activated CD4^+^ T cell populations (Supplemental Figure 8, D and E). AdultTRMs had a significant increase in Tbet MFI and Tbet^+^ percentage in activated T cells compared with NeoTRMs and host CD45.1^+^ T cells (Supplemental Figure 8, F and G). In mice receiving AdultTRMs, the host CD45.1^+^ CD4^+^ T cells had increased Tbet compared with host cells from mice receiving NeoTRMs, suggesting transfer of these cells affected secondary responses (Supplemental Figure 8, F and G). No mucus production was observed in this model.

Together, these findings indicate that TRMs generated after neonatal HMPV infection are both necessary and sufficient to induce asthma pathology upon rechallenge.

### JAK2 inhibition early in reinfection mitigates asthma pathology.

**Figure 7:**
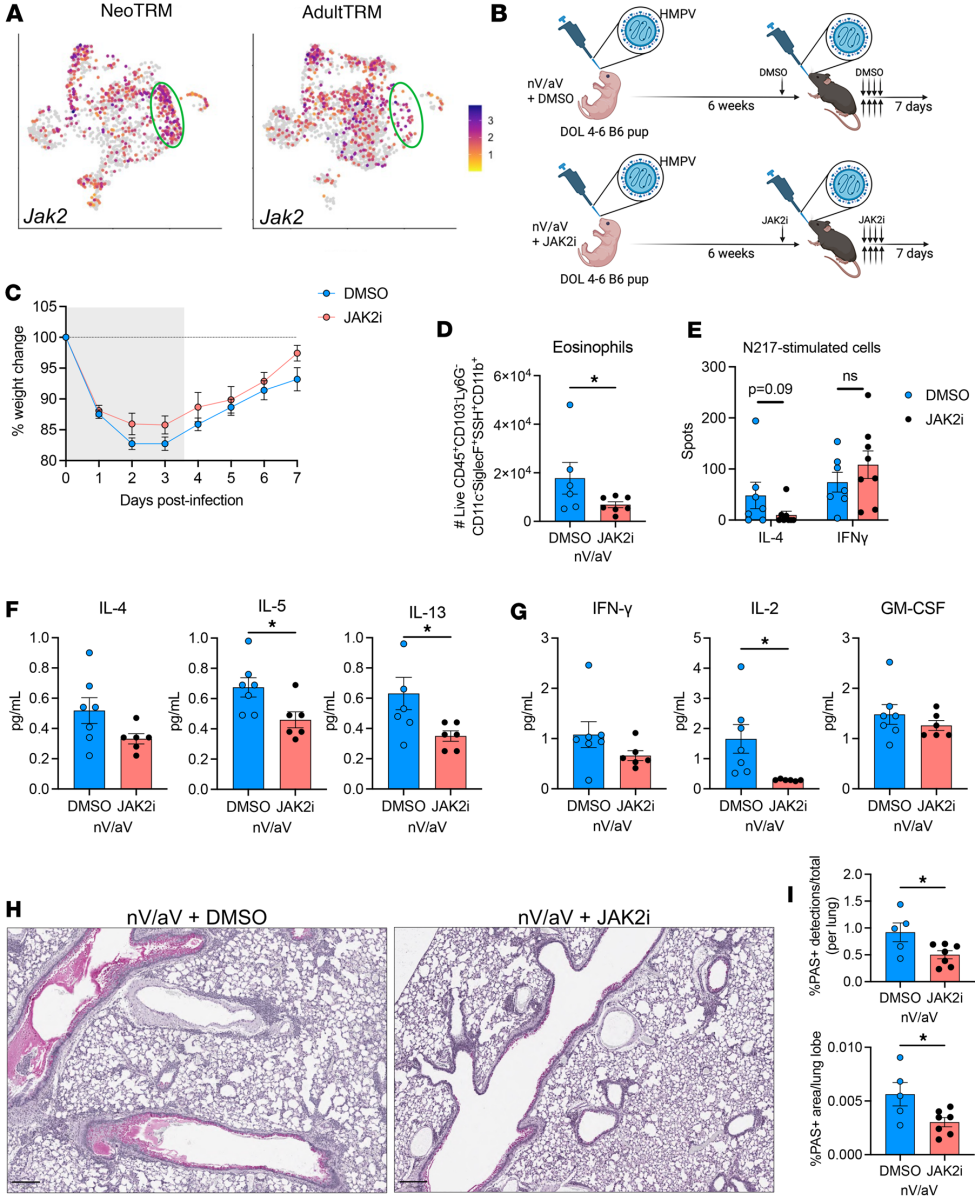
Early JAK2 inhibition reduces asthma pathology. (**A**) UMAP visualization of *Jak2* expression, showing upregulation in green circle corresponding to the Th2 cluster. (**B**) Mice were treated with fedratinib (120 mg/kg) once prior to rechallenge followed by BID dosing for the first 4 days after rechallenge. DMSO was used for vehicle control treatment. Created with BioRender.com. (**C**) Weight changes after rechallenge with HMPV. (**D**) Quantification of eosinophils 7 days after rechallenge. *P < 0.05 by Mann-Whitney *U* test. (**E**) N217-specific IL-4 and IFN-γ production after HMPV rechallenge, with a trend (*P* = 0.09, 2-tailed Student’s *t* test) toward reduced IL-4 production in JAK2i-treated mice. (**F** and **G**) IL-4, IL-5, IL-13, IL-2, IFN-γ, and GM-CSF protein quantity from whole lung homogenate. (**H**) Representative images of PAS staining demonstrating mucus production in large airways with reduction in JAK2i. Scale bar: 250 μm. (**I**) Quantification of PAS^+^ staining as percentage of total cell detections (left) and lung area (right). *P < 0.05 by 2-tailed Student’s *t* test.

Broad CD4-depletion methods ameliorated asthma pathology, so we next sought to identify Th2 TRM-specific targets to assess novel therapeutic strategies. After examining the top cluster-defining genes (Figure 4A), we identified that *Jak2* was significantly upregulated in the Th2 cluster (Figure 7A). JAK2 is a member of the JAK/STAT signaling cascade downstream of numerous cytokine pathways and relatively specific inhibitors of individual JAK proteins exist clinically. We treated mice with fedratinib, a selective JAK2 inhibitor (JAK2i), early in the nV/aV model (Figure 7B). Mice treated with JAK2i had a trend in improved weight gain after reinfection (Figure 7C). At day 7 after infection, eosinophil number in the lung was reduced (Figure 7D). Antigen-specific IL-4 production was largely undetectable in the JAK2i-treated mice (*P* = 0.09), and antigen-specific IFN-γ production was unchanged (Figure 7E). Similarly, Th2 cytokines in the lung homogenate were reduced in JAK2i-treated mice (Figure 7F). IL-2, a cytokine with differential expression in the Th2 cluster, also showed significant reduction (Figure 7G). However, there was no significant difference in IFN-γ or GM-CSF quantity with JAK2i-treatment (Figure 7G). Mucus production in the large airways was reduced in JAK2i-treated mice (Figure 7, H and I). Collectively, these studies offer a proof-of-concept that targeted intervention directed at a specific subset of TRMs, in this case Th2, can be effective in mitigating pathological responses with secondary challenge.

## Discussion

The current study demonstrates that TRMs can contribute to development of asthma-like inflammation after viral rechallenge. Enhancement, targeted depletion, and transfer of TRMs demonstrate a role for this small population in asthma pathology. Th2 TRMs were almost exclusively formed after neonatal infection, clonally expanded, and produced cytokines (e.g., *Il4*) associated with disease. Taken together, this model recapitulates the clinical phenomenon of early-life viral infection conferring an increased risk of developing asthma, with identification of a potentially targetable subpopulation of Th2 TRMs.

TRMs are established after early life exposures and fill a niche in the lung, but may have different functional capabilities. In an animal model of influenza infection, infant mice generated fewer TRMs compared with adult mice (60), similar to reduced capabilities of neonates infected with HMPV (Figure 3B). Moreover, the TRMs formed to early influenza infection were less likely to provide protection upon rechallenge with a heterotypic strain (60). A study using human cadaveric specimens found that memory CD4^+^ and CD8^+^ T cells are more abundant in the human lung after 2 years of age when compared with naive cells (61). Lung CD4^+^ TRMs from children under 3 had a distinct transcriptional profile and reduced functionality (defined as IFN-γ or TNF-α production) compared with memory cells from older children (61). Clonotype profiles of human CD4^+^ TRMs in the lung were site specific, showing minimal overlap with other mucosal sites, with stable frequencies of expansion over time (61). Interestingly, a type 2 signature was not observed in early life in human lung memory CD4^+^ T cells by bulk RNA-seq, although early-life viral infection status of the individuals was not known (61). The current study demonstrates that TRMs represent a heterogenous group, with pathological Th2s in our system representing approximately 20% of the total population after neonatal HMPV (Figure 4A).

Despite the small frequency of an even smaller TRM population, the Th2-skewed TRMs formed after neonatal infection appear to be contributing to asthma pathology. In humans, a similar lung-resident Th2 population defined by *GATA3*, *IL4*, *IL5*, and *IL13* expression was enriched in patients with asthma compared with healthy controls (62). The single-cell approaches further demonstrated enhanced markers of Th2 pathogenicity (e.g., *IL17RB*, *PPARG*) and uncovered an epithelial cell–TRM communication network that may be a pivotal contribution to asthma pathology (62, 63). In animal models of allergen sensitization, Th2-skewed TRMs are prolific early producers of cytokines and are sufficient to cause airway hyperresponsiveness after rechallenge (64, 65). Similar pathogenic roles are noted for TRMs formed after neonatal HMPV infection, highlighting the temporal importance of early life exposures in biasing this stable memory population in the lung.

In addition to TRMs, the immunological connection between early-life viral infection with recurrent wheeze likely requires multiple cell types and pathways. Early-life RSV infection leads to the release of the epithelial cell–derived cytokine IL-33, promoting the expansion of ILC2s (59). Blockade of IL-33 or genetic ablation of IL-33 signaling (e.g., *Il1rl1^–/–^*) led to improved airway hyperresponsiveness and eosinophil recruitment, in part by reducing ILC2 and Th2 frequency (59). A similar pathway of IL-33 and ILC2 expansion was observed in human infants with severe RSV infection (66). IL-33 signaling was dispensable in the context of HMPV rechallenge, suggesting differential or redundant upstream cytokines may be contributing to these model systems. In addition to IL-33, the upstream epithelial alarmin TSLP may play in a role in connecting viral infections and asthma. Asthma pathology after early-life RSV infection, particularly in male mice, was abrogated in mice lacking *Tslpr*, the receptor for TSLP (67). The nature and directionality of the relationship between epithelial cytokine production and TRM activation could be further explored in these animal models of early-life virus-induced asthma.

The clinical studies of asthma development clearly identify a pattern of increased risk following a significant early-life viral infection (34–39). These findings are supported by the animal models, in which an early life exposure to a virus (including RSV, rhinovirus, and now HMPV) can alter secondary responses to contribute to asthma-like responses (67–69). Interestingly, the enhanced Th2-skewing extends beyond the viral response, as early-life respiratory infection enhances allergic responses (67, 68, 70–73). Although antigen-specific by nature, TRMs may play a role in this process either by shaping de novo T cell responses and/or by functioning as sentinels providing an early bystander response to a new exposure (74–76). The role of virus-specific lung TRMs or the overall milieu they help form in shaping subsequent responses to exposures could be highly informative as it relates to these findings.

Given the role TRMs play in a model of early-life viral infection–induced asthma, a therapeutic strategy would be to target the subset of TRMs that contribute to pathology while leaving protective memory unperturbed. In this study, use of a JAK2 inhibitor, as directed by single-cell RNA-seq analysis, provides proof of concept that this approach could be feasible. JAK2 is downstream of several stimuli, including IL-12, IL-23, IFN-γ, gp130 family members, IL-5, erythropoietin, and growth hormone (77). JAK2 associates with STAT5, usually found in regulatory T cells, and STAT6, a canonical Th2 transcription factor (78). One study demonstrated that fedratinib treatment significantly reduced STAT6 phosphorylation/activation, which could explain abrogated Th2 pathology in our system. Interestingly, JAK2i did not alter IFN-γ production in this system, demonstrating at least some degree of specificity by this approach. Clinically, JAK2 mutations are often associated with myeloproliferative neoplasms or myelofibrosis, although pan-JAK and JAK1/3 inhibitors are being evaluated in asthma (78, 79). Selective JAK2 inhibitors may also be limited in the context of pediatric asthma or virus-induced wheeze, as fedratinib treatment has been associated with significant toxicities, such as anemia, gastrointestinal side effects, and rare cases of encephalopathy (80). It is not clear what signal induces upregulation of *Jak2* in the pathological Th2 TRMs in this system, but future studies could investigate candidate cytokines as other therapeutic avenues. Additionally, future studies could evaluate conditional JAK2 deletion in CD4^+^ T cells to definitively demonstrate that this is a CD4-intrinsic process. The approach of targeting Th2 TRMs would potentially be promising, given the generalizability of the findings of early-life viral infection and asthma, although more work will be needed to clarify Th2 TRM formation in response to other stimuli.

The current study has limitations, which could be addressed in future studies. First, depletion of TRMs in the lung can be problematic for a number of reasons, including the small population size and targeting of the depletion mechanism to the local environment. Treatment of an anti-CD4 antibody directly to the airway, for example, has been shown to adequately deplete airway but not parenchymal or vascular CD4^+^ T cells (81). This approach is limited by depletion of all T cell subsets and by potentially leaving a residual subset of lung TRMs not associated with airways; this is one hypothesis for the partial phenotype observed after o.p. anti-CD4 in this study. Further, use of JAK2 inhibition may have effects outside of TRMs, such as that shown in prior work that demonstrated fedratinib can induce eosinophil apoptosis (82). Unlike the mixed virus/allergen models described above, the current model uses two exposures to the same virus, varying the time of initial exposure. Future studies could build on this work by evaluating mixed viral challenges (e.g., HMPV then RSV) or by adding sensitization to common allergens (e.g., house dust mite) to understand the necessity of cognate antigen in activation of TRMs. Repetitive exposure to an antigen has been shown to induce neutrophilic lung inflammation, effectively modeling the emerging Th2-low asthma endotype observed in pediatric patients (9, 83). Understanding the underlying TRM contributing to pathology therefore becomes imperative, as this phenotyping could inspire targeted therapeutic intervention. However, TRMs remain a challenge to isolate and characterize from patients, particularly as invasive procedures are seldom performed in small children. The differential TRMs formed after exposure in neonates versus adults could inspire future investigation into the duration/longevity of these cells after an early life exposure. The current study demonstrates the importance of these lung-resident cells in asthma pathology after viral exposure early in life, adding to our understanding of the immunological mechanisms linking these two common pediatric pathologies.

## Methods

### Sex as a biological variable.

Both male and female mice were used throughout the experiments.

### Mice and virus stocks.

Six- to 8-week-old C57BL/6 mice (strain 000664) and B6.SJL-*Ptprc^a^ Pepc^b^*/BoyJ mice (strain 002014, referred to as CD45.1 B6) were purchased from The Jackson Laboratory. *Il1rl1^–/–^* mice were provided in-house. Breeder pairs were established under BSL-2 conditions. Pups were infected on days 4–6 with 2.8 × 10^6^ PFU of HMPV strain TN/94-49 (genotype A2) in 10 μL sterile PBS after isoflurane anesthesia in a heated chamber, as previously described (54). Adult (6–8 weeks) C57BL/6 mice were o.p. inoculated with 2.8 × 10^6^ PFU of HMPV in 100 μL sterile PBS (84, 85). For reinfection, mice were anesthetized and o.p. inoculated with 1.0 × 10^7^ PFU of HMPV in 100 μL sterile PBS (55). To generate UV-inactivated HMPV, the above viral stocks were placed in a sterile 6-well dish and placed in the Stratagene 1800 UV-Stratalinker on automatic cross-linking (1,200 × 100 μJ) for three 10-minute intervals. UV-inactivated virus was titered via plaque assay to ensure no replicating virus was present. Bronchoalveolar lavage was performed as previously described (86). Daily weights were recorded, and mice were euthanized if weight reached less than 70% of starting body weight. Neonatal mice were euthanized via decapitation; adult mice were euthanized via CO_2_ asphyxiation. HMPV was grown in LLC-MK2 cells and purified as previously described (87).

### Luminex.

The right lower lobe of lung was homogenized, clarified, and diluted per the manufacturer’s instructions (Invitrogen, ProcartaPlex assay).

### Flow cytometry.

Lung tissue was minced and chemically digested using DNase/collagenase for 1 hour at 37°C. Tissue was then passed through a 70 μm strainer and treated with ACK RBC lysis buffer (Gibco, A1049210) for 2 minutes. After centrifugation for 5 minutes at 500 × *g* and resuspension, the cells were in a single-cell suspension and aliquoted into various panels as below (Supplemental Table 1).

For adaptive (Supplemental Figure 9) and innate (Supplemental Figure 10) panels, cells were plated in a 96-well V-bottom plate and stained with live/dead violet (1:1,000 in PBS, Invitrogen, L34964A) for 15 minutes at room temperature. Cells were washed twice in FACS and treated with Fc block (1:100 in FACS buffer, Tonbo, 70-0161-M001). After removal of Fc block, cells were stained for surface markers (1.5 μL antibody/sample in BD Horizon buffer, 566349) for 45 minutes at 4°C. After 2 washes, cells were fixed using FOXP3 fix/perm buffer overnight for 18 hours (Invitrogen, 50-112-8857). After a wash in perm buffer, cells were stained for intracellular markers (2.5 μL transcription factor antibody/sample in 1:1 mixture of BD buffer/perm buffer) for 1 hour at 4°C. Cells were washed twice in perm buffer, resuspended in FACS, and quantified using counting beads.

For stimulation assays, cells were plated in a 96-well, U-bottom plate and treated with either 10 μM HMPV N217 (a class II restricted epitope) or an irrelevant peptide (GP66-77 from LCMV) (53). BFA/Monensin was used to inhibit cytokine release. CD107-PE was added to assess degranulation. Unstimulated and PMA/ionomycin (eBioscience, 00-4970-03) conditions were used for controls. Cells were incubated for 5 hours at 37°C. After stimulation, live/dead, Fc block, and surface staining were performed as above. After a 20-minute fixation, cells were stained for cytokine production (4.5 μL antibody/sample).

For both assays, staining was completed in the dark. Cells were strained through nylon filters and analyzed on a Cytek Aurora multispectral flow cytometer. Unstained fixed cells were used for unmixing. FMO controls or isotype controls were used for gating placement and are presented when applicable. Data analysis was performed using FlowJo (v.10.10.1).

### Airway hyperresponsiveness measurements.

Mice were anesthetized (90 mg/kg i.p. pentobarbital), tracheotomized, and mechanically ventilated at 150 breaths/min with a tidal volume of 10 mL/kg and positive end expiratory pressure of 3 cmH_2_O using a computer-controlled small-animal ventilator (FlexiVent; SCIREQ) as previously described (88, 89). Broadband frequency forced oscillation techniques (Quick Prime-3) were used to establish a baseline, followed by inhalation of increasing doses of aerosolized methacholine (0–50 mg/mL). The highest measurement within the first 10 perturbations after methacholine treatment was recorded.

### Histology.

Lungs were fixed with 10% formalin, embedded in paraffin, and stained with H&E or periodic acid–Schiff (PAS) stain. PAS quantification was performed using QuPath. Lung tissue was outlined, and cellular detection was performed. Object classification was then performed on a representative slide to identify approximately 30 PAS-positive structures and 30 PAS-negative structures. Cell number, area, and PAS-positive detections were then calculated on individual batches in a blinded manner (Supplemental Figure 11).

### RNAscope.

Lungs were fixed in 10% formalin, embedded in paraffin, and sectioned. Lung tissue slides were deparaffinized by incubating at 60°C in HbEZ II hybridization oven (ACD), followed by incubating in xylene for 5 minutes twice, then incubating in ethanol for 2 minutes twice. Hydrogen peroxide was added to slides at room temperature for 10 minutes before a target retrieval step was performed by putting slides in target retrieval buffer that was heated to 98°C for 15 minutes. RNA probes provided by the manufacturer were added to samples, amplified, and hybridized to fluorophores at 40°C as per the manufacturer’s instructions for RNAscope Multiplex Fluorescent v2 Assay (ACD, 323270). The following probes from ACD were used: *Cd4* (406841, channel 1), *Gata3* (403321, channel 3), *Cd19* (314711, channel 2). Counterstain was performed with DAPI as provided in the manufacturer’s kit. Images were captured on a Leica Stellaris 5 confocal microscope and processed using Fiji v2.16.

### ELISpot.

After generation of a single-cell suspension, 50,000 cells/well were plated and stimulated with either 10 μM irrelevant GP66-77 peptide or N217 (53). After 48 hours at 37°C, plates were processed per the manufacturer’s instructions (R&D Systems, EL404 and EL405).

### TRM staining.

Exclusion of intravascular cells was performed via tail vein injection of 15 μg of anti-CD45 antibody with euthanasia 3 minutes after injection (56). Lung tissue was then processed and stained for flow cytometry as above.

### Single-cell RNA-seq.

After intravascular labeling and surfacing staining including cell hashing antibodies (BioLegend), TRMs were isolated via flow cytometry sorting with gating using intravascular CD45 exclusion and CD19^–^CD3^+^CD4^+^. Cells were then passed through a 40 μm strainer, enumerated by Cellometer 2000 (Nexcelom), and loaded onto 10x Genomics Chromium controller for cell capture using the 5′ V2 kits (84). Libraries for gene expression and hashtag oligos were constructed following protocols from 10x Genomics and the New York Genome Center. Final libraries were quality-controlled by Agilent TapeStation, then sequenced on an Illumina NovaSeq 6000 targeting 50,000 reads per cell. Sequencing data were processed with CellRanger 7.0 with downstream analysis via Seurat 4.0 using R (version 4.1.1). Quality control metrics included nFeature_RNA>200, nFeature_RNA<5000, and percent.mt<20 followed by normalization. Samples were then demultiplexed by hash-tag-oligos (HTOs), and doublets identified by HTOs were removed before downstream analysis.

### Antibody-mediated CD4 depletion.

Anti-CD4 (clone GK1.5, BioXcell) or isotype control was given via i.p. injection (500 μg) or via o.p. administration (100 μg) to anesthetized mice 1 day prior to rechallenge.

### FTY720/fedratinib treatment.

FTY720 was resuspended in 100% ethanol and diluted in peanut oil to a concentration of 5 mg/mL. Mice were treated with 50 μg/dose diluted in peanut oil or vehicle control via i.p. injection. Treatment was initiated 2 days prior to reinfection. Fedratinib (MCE, HY-10409) was resuspended in DMSO, diluted 1:10 in corn oil, and administered at 120 mg/kg BID via oral gavage. Fedratinib treatment was started with a single dose prior to reinfection and continued until day 3 after infection.

### TRM adoptive transfer.

Neonatal or adult CD45.2 B6 mice were infected with HMPV and aged for 6 weeks. Lung tissue was isolated and CD4^+^ T cells were isolated from a single-cell suspension using magnetic bead positive selection (Miltenyi Biotec, 130-117-043) per the manufacturer’s instructions. Naive CD45.1 B6 adults were then challenged with HMPV followed by adoptive transfer of 5 × 10^5^ of either AdultTRMs or NeoTRMs (90).

### Statistics.

All data are displayed as mean ± SEM. All statistical analysis was performed using GraphPad Prism for Mac (v.10.4.2). Analyses with 2 groups were analyzed using an unpaired 2-tailed Student’s *t* test; 3 or more groups were analyzed using a 1-way ANOVA with Dunnett’s multiple-comparison test unless otherwise specified. Experiments with 2 groups and multiple conditions were analyzed using a 2-way ANOVA with Tukey’s multiple-comparison test. Significance was defined as a *P* value less than 0.05 for all analyses.

### Study approval.

The studies were approved by the University of Pittsburgh IACUC (protocol 23053068).

### Data availability.

Numerical data underlying figures are reported in the Supporting Data Values file. Flow cytometry data (both raw and unmixed files) will be made available upon request. Single-cell RNA-seq data are deposited in NCBI’s Gene Expression Omnibus (GEO GSE320064). Code is available at https://github.com/eddenstj/Neonatal-TRMseq (commit ID 4cc06dc).

## Author contributions

All authors provided critical contributions to this manuscript. EEB, JL, OBP, and TE designed research studies, conducted experiments, acquired data, and wrote the manuscript. EEB and JL share first authorship; order was assigned using alphabetical order of the last name. LF, DL, KC, AS, AM, AJW, and MLM acquired data. LM and HRT provided reagents. All authors contributed to revising the manuscript through their careful review.

## Conflict of interest

The authors have declared that no conflict of interest exists.

## Funding support

This work is the result of NIH funding, in whole or in part, and is subject to the NIH Public Access Policy. Through acceptance of this federal funding, the NIH has been given a right to make the work publicly available in PubMed Central.

- NIH National Heart, Lung, and Blood Institute, 1F30HL159915 (to OBP).
- AAAAI Foundation Faculty Development Award (to TE).
- NIH National Institute of Allergy and Infectious Diseases, K08AI182486 (to TE).
- NIH Eunice Kennedy Shriver National Institute of Child Health and Human Development under Award Number K12HD000850 (to TE).
- Shear Family Foundation.

## Supplementary Material

Supplemental data

Supporting data values

## Version 1 03/10/2026

In-Press Preview

## Version 2 04/22/2026

Electronic publication
