# Supplementary material for: Early-life viral infection generates pathological tissue-resident memory cells that contribute to asthma-like disease
Source: JCI Insight. 2026 Mar 10;11(8):e198712. doi: 10.1172/jci.insight.198712 (PMC13135394; doi:10.1172/jci.insight.198712)
Supplement: Supplemental data [file jciinsight-11-198712-s273.pdf]

Title: Early life viral infection generates pathologic tissue resident memory cells that contribute to asthma-like airway disease

Emma E. Brown*[1], Jie Lan*[1], Olivia B. Parks[2], Li Fan[3], Dequan Lou[3], Alysia McCray[1], Lisa Mathews[4], Alexander J. Wardropper[5], Anna Shull[6], Michelle L. Manni[7], Heth R. Turnquist[4], Kong Chen[3], Taylor Eddens[1,8,†]

[1]University of Pittsburgh School of Medicine, Department of Pediatrics, Pittsburgh, PA, USA

[2]University of Pittsburgh Medical Scientist Training Program, Pittsburgh, PA, USA

[3]University of Pittsburgh School of Medicine, Department of Medicine, Pittsburgh, PA, USA

[4]University of Pittsburgh School of Medicine, Department of Immunology and Starzl Transplant Institute, Pittsburgh, PA, USA

[5]Washington and Jefferson College, Washington, PA, USA

[6]University of South Carolina, Columbia, SC, USA

[7]University of Pittsburgh School of Medicine, Department of Pharmacology and Chemical Biology, Pittsburgh PA, USA

[8]Institute for Infection, Inflammation, and Immunity in Children (i4Kids), Pittsburgh, PA, USA

*denotes co-first authors, [†]denotes corresponding author

Corresponding Author:

*Taylor Eddens

4401 Penn Avenue

Rangos Research Building 9128

Pittsburgh, PA 15224

Phone: 724-272-9208

Email: taylor.eddens@chp.edu

Supplemental material:
Supplemental Table 1
Supplemental Figures 1-11

Supplemental Table 1. FACS panel reagents.

Adaptive panels:	Fluorophore	Species	Catalog Number	Clone
CD19	BV785	Rat	115543	6D5
CD3e	BUV395	Hamster	565992	145-2C11
CD4	AF700	Rat	100536	RM4-5
CD44	APC-Cy7	Rat	560568	IM7
CD62L	BUV563	Rat	741230	MEL-14
CD8a	AF532	Rat	58-0081-80	53-6.7
CD90.2	PE-Cy7	Rat	561642	53-2.1
CD90.2 (alternative)	BUV661	Rat	741457	53-2.1
ST2	BUV805	Rat	749322	U29-93
CD11a	BV510	Rat	747760	M17/4
CD69	AF647	Hamster	104518	H1.2F3
CXCR6	APC	Rat	151106	SA051D1
CD45.2 (i.v. labeling)	BUV496	Mouse	741092	UV7
Foxp3	PerCP-Cy5.5	Rat	45-5773-82	FJK-16s
T-bet	PE	Mouse	644809	4B10
GATA-3	BV711	Mouse	565449	L50-823
TCF1	BV421	Mouse	566692	S33-966
Peptide stimulation plate				
IFNg	BV650	Rat	505831	XMG1
IL-4	BV711	Rat	564005	11B11
IL-5	APC	Rat	554396	TRFK5
CD107	PE	Mouse	121612	1D4B
IL-13	PE-Cy7	Rat	25-7133-82	eBio13A
Innate panel				
CD45.2	BUV496	Mouse	741092	UV7
Ly-6G	APC-Cy7	Rat	127624	1A8
Ly-6C	AF700	Mouse	128024	HK1.4
CD103	BV785	Rat	121439	10F.9G2
CD11b	APC	Rat	101211	MI/70
CD11c	BUV805	Hamster	749090	HL3
F4/80	BV421	Rat	123131	BM8
SiglecF (CD170)	AF647	Rat	155519	S17007L

Supplemental Figures

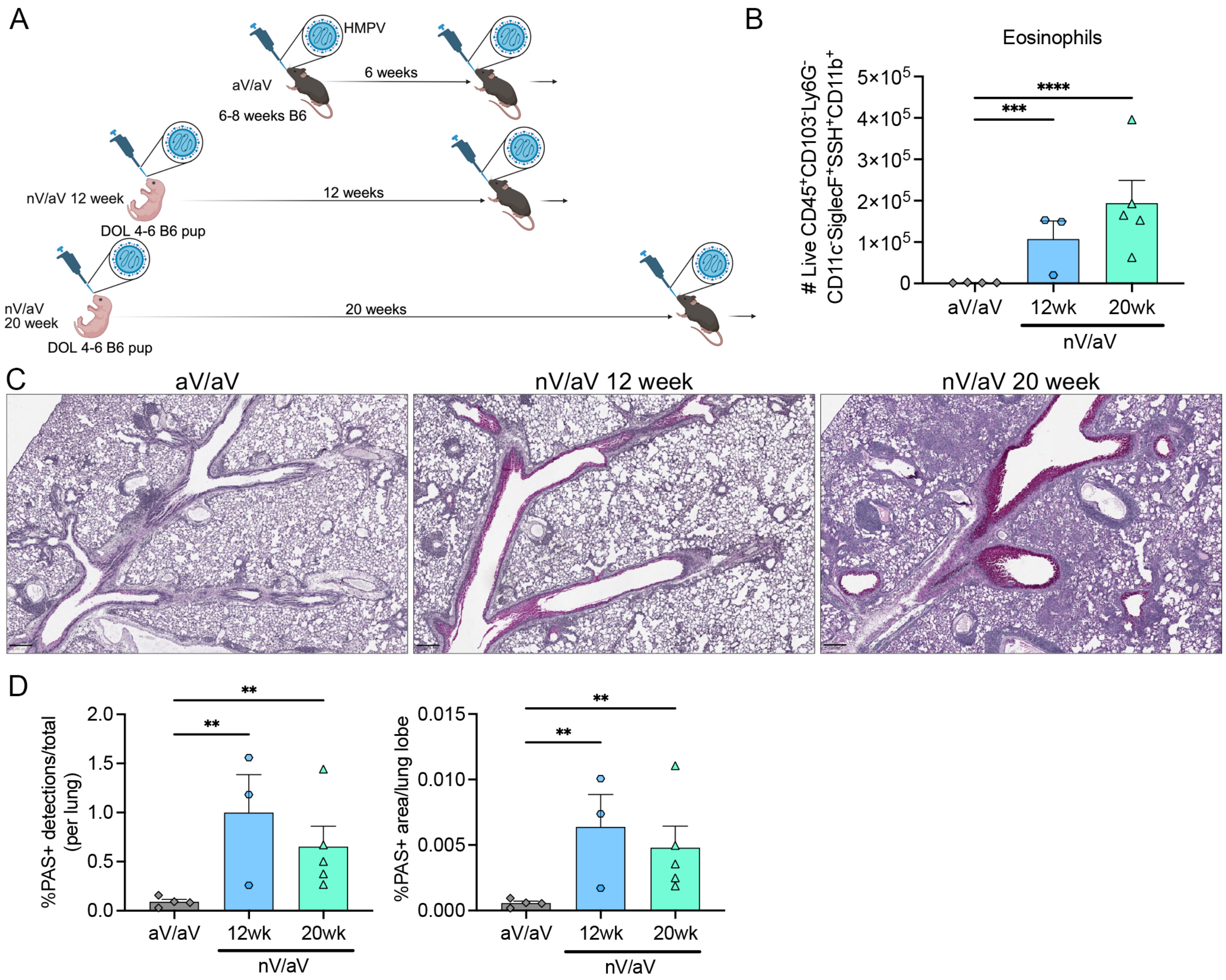

Supplemental Figure 1. **nV/aV infection demonstrates asthma pathology if age-matched with aV/aV model**. **A**.) Schematic of model showing 12 week nV/aV model aligns with age of aV/aV mice at time of sacrifice and 20-week extension of the nV/aV model. Created with Biorender.com. **B**.) Eosinophil recruitment 7 days after rechallenge with HMPV. *p<0.05 by one way ANOVA with Dunnett multiple comparisons testing. **C**.) PAS staining demonstrating mucus production. Scale bar= 100µm. **D**.) Quantification of PAS+ staining as percentage of total cell detections (left) and lung area (right). *p<0.05, **p<0.01 by student's t-test.

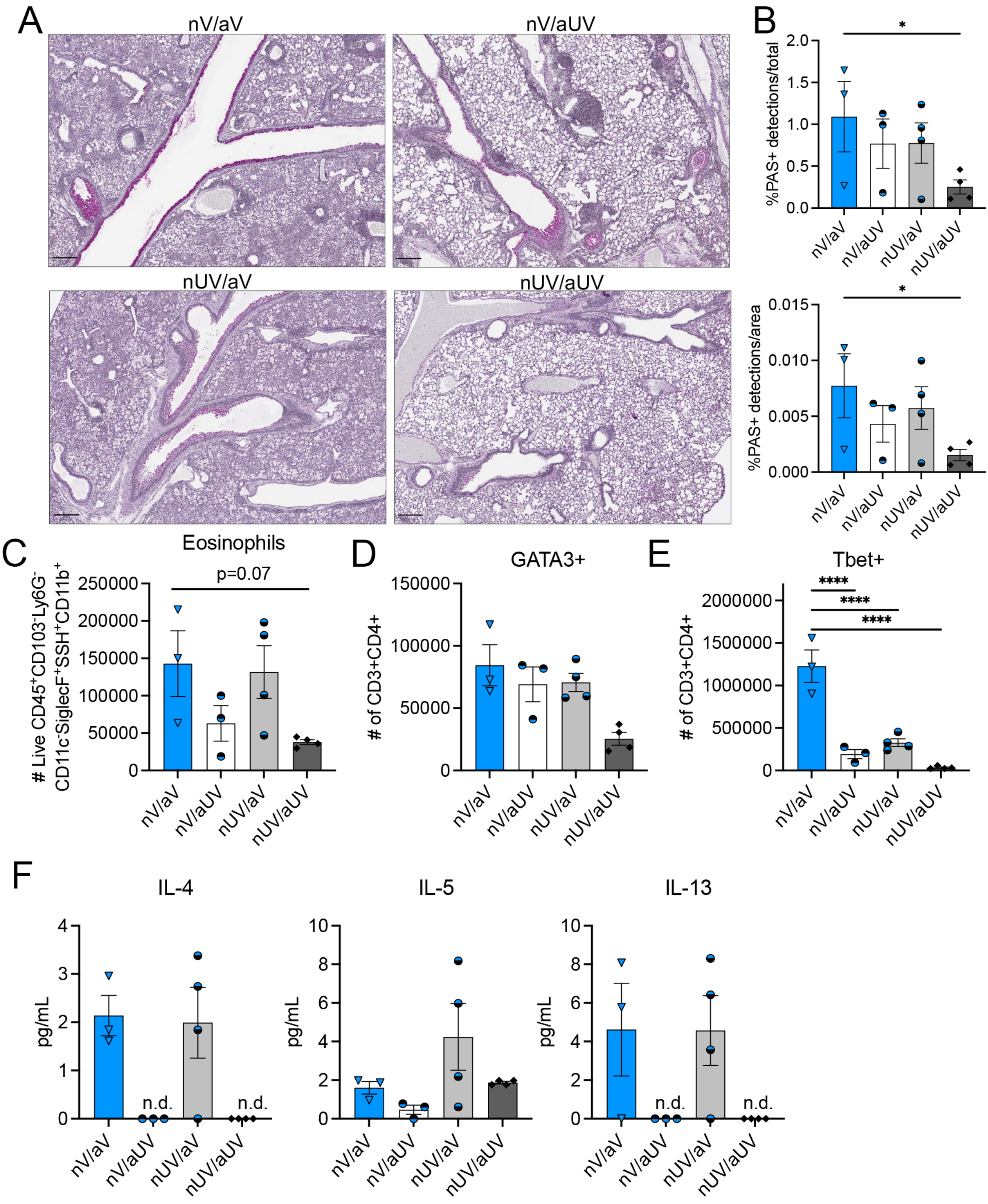

Supplemental Figure 2. **UV-inactivated HMPV exposure can still contribute to pathology in rechallenge model**. Mice were treated with HMPV or UV-inactivated virus as neonates (nV or nUV, respectively) and rechallenged in adulthood with HMPV or UV-inactivated virus (aV or aUV). **A**.) nV/aUV and nUV/aV groups had similar PAS+ mucus induction and inflammation as nV/aV mice. **B**.) Quantification of PAS+ staining as percentage of total cell detections (top) and lung area (bottom). **C**.) nV/aV and nUV/aV treated mice had similar number of eosinophils. p=0.07 by one-way ANOVA with multiple comparisons. **D**.) Similar GATA3 CD4$^+$ T cell

number between nV/aV, nV/aUV, and nUV/aV groups. **E**.) nV/aV increased $Tbet^+$ cell number compared to other groups. ****$p<0.0005$ by one-way ANOVA with multiple comparisons. **F**.) Type 2 cytokine production with detectable IL-4 and IL-13 in nV/aV and nUV/aV groups only.

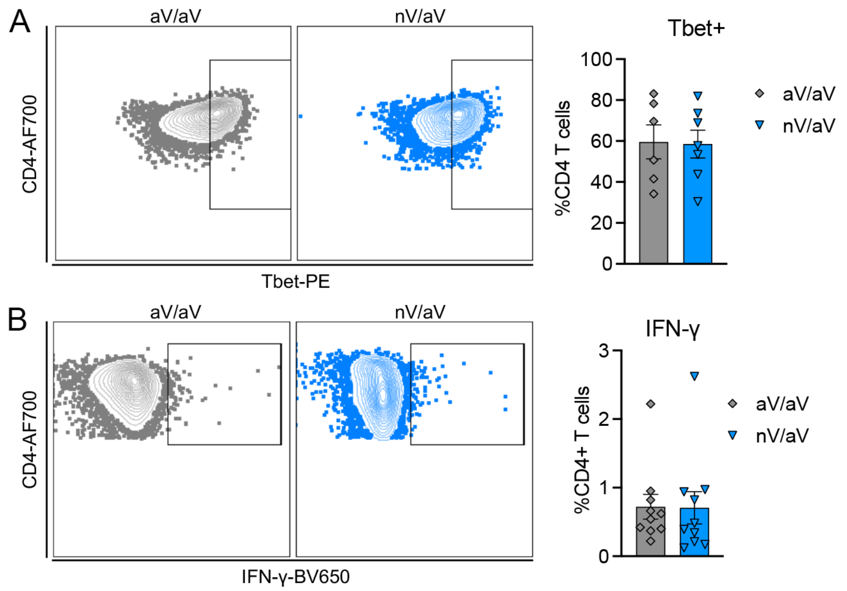

Supplemental Figure 3. Similar Th1 responses in nV/aV and aV/aV models.

A.) Representative flow plots and quantification of intracellular Tbet staining in $CD4^+$ T cells isolated from the lung at day 7 post re-challenge. **B**.) Intracellular staining of IFN-γ production following *ex vivo* HMPV peptide stimulation in lung $CD4^+$ T cells 7 days post re-challenge.

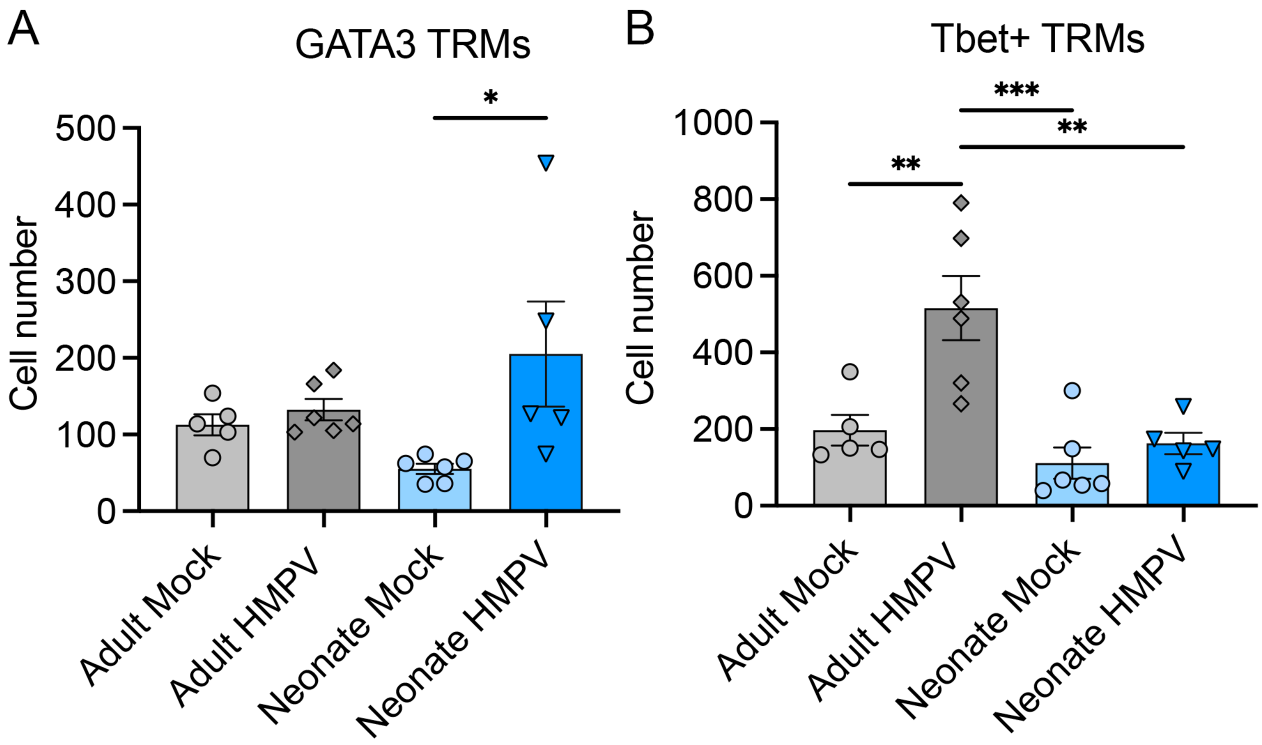

Supplemental Figure 4. **Quantification of Th2 and Th1 TRMs**. **A**.) Quantification of $GATA3^+$ TRMs 35-days after neonatal or adult mock or HMPV infection. **B**.) Quantification of $Tbet^+$ TRMs 35-days after neonatal or adult mock or HMPV infection. *p<0.05, **p<0.01, ***p<0.005 by one-way ANOVA with multiple comparisons.

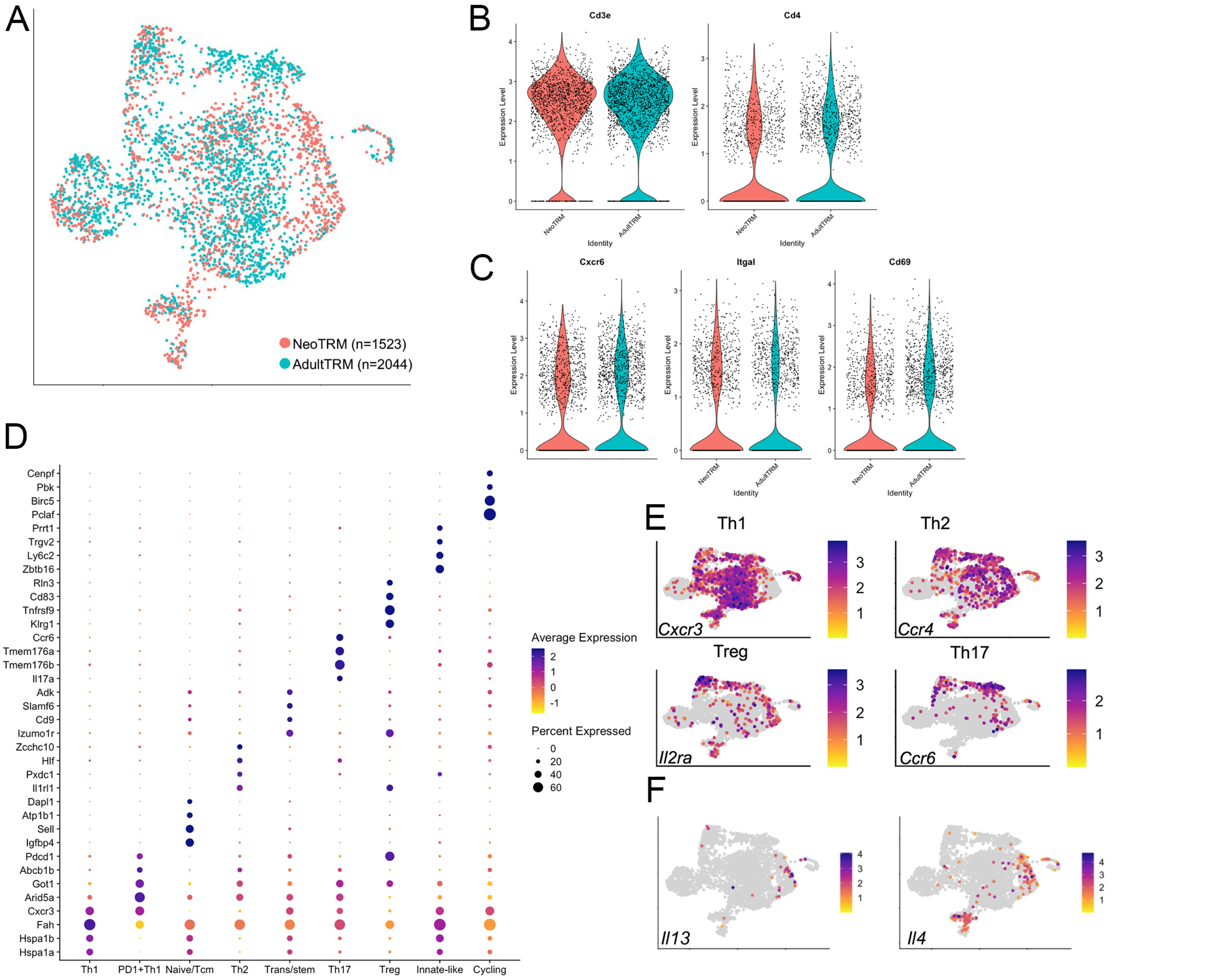

Supplemental Figure 5. **Single cell RNA sequencing of tissue resident memory cells, cluster identification, and chemokine receptor expression**. **A**.) UMAP visualization of sequenced TRMs isolated 5 weeks following neonatal (NeoTRM, red) or adult (AdultTRM, blue) HMPV infection. **B**.) Expression of *Cd3e* and *Cd4* in NeoTRM and AdultTRM. **C**.) Expression of TRM markers, including *Cxcr6*, *Itgal* (CD11a), and *Cd69*. **D**.) The top 4 differentially expressed genes per Seurat cluster, which were renamed as described in the text. **E**.) Chemokine receptor (*Cxcr3*, *Ccr4*, and *Ccr6*) and *Il2ra* expression corresponding to individual CD4^{+} T cell subset. **F**.) *Il13* and *Il4* expression demonstrating Th2 effector cytokine production.

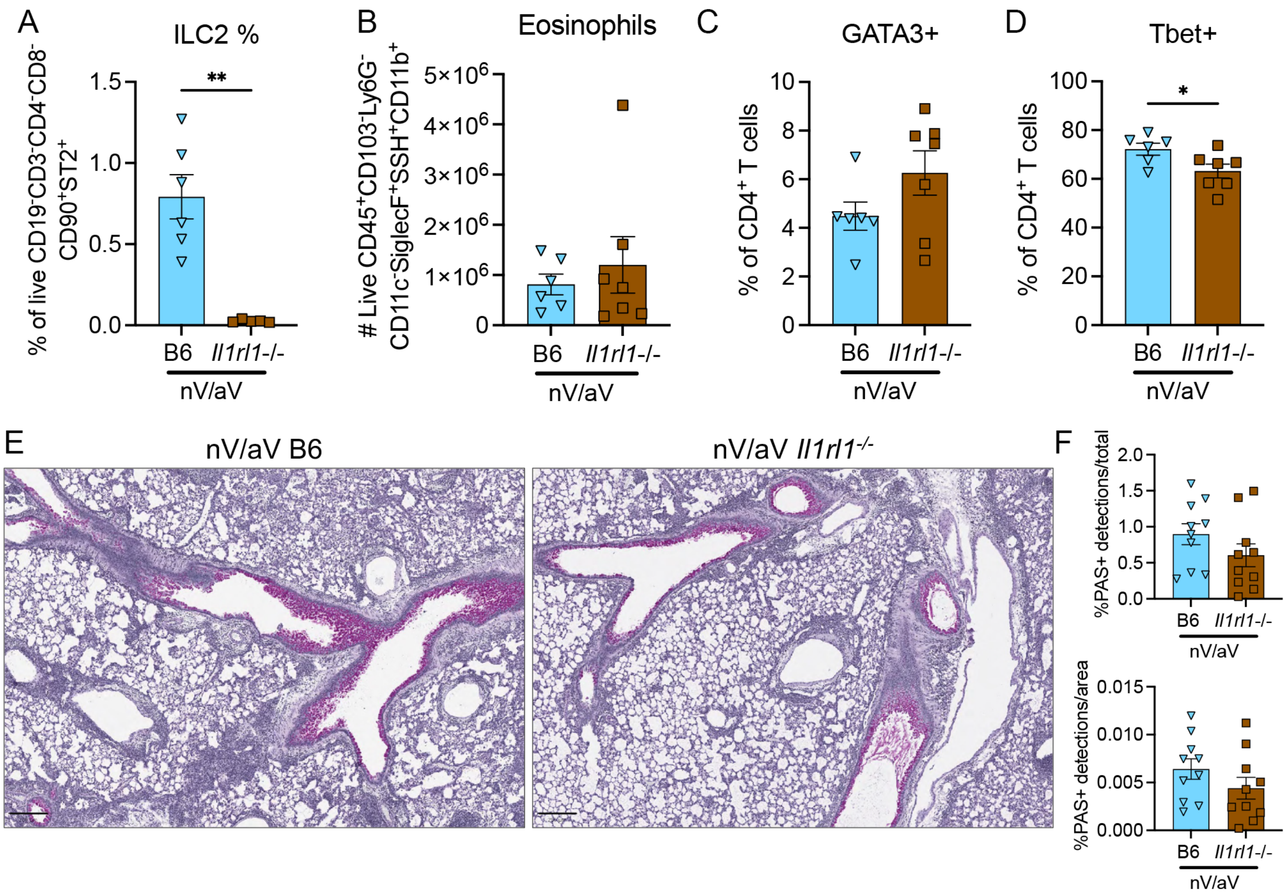

Supplemental Figure 6. **IL-33 signaling is dispensable for nV/aV pathology**. B6 or *Il1rl1*$^{-/-}$ mice were infected in nV/aV model. **A**.) Frequency of ILC2s was significantly reduced in *Il1rl1*$^{-/-}$ mice. *p<0.01 by student's t-test. **B**.) Similar number of eosinophils in B6 and *Il1rl1*$^{-/-}$ mice. **C-D**.) Frequency of GATA3^{+} and Tbet^{+} CD4^{+} T cells at day 7 post-nV/aV. *p<0.05 by student's t-test. **E-F**.) PAS+ mucus production was similar between B6 and *Il1rl1*$^{-/-}$ mice.

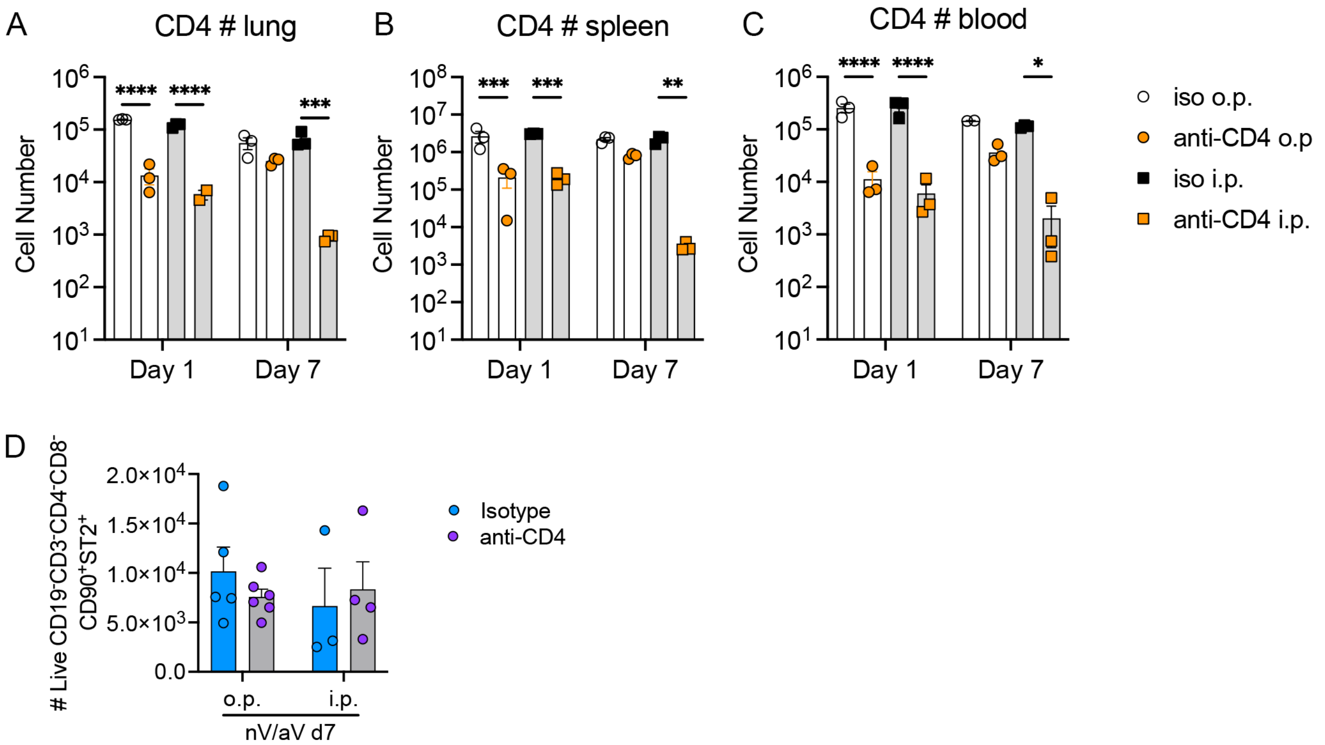

Supplemental Figure 7. Anti-CD4 depletion via o.p. and i.p. routes leads to differential CD4$^+$ T cell recovery and does not change ILC2 number. A-C.) Time course of CD4$^+$ T cell recovery from lung (A), spleen (B), and peripheral blood (C) following treatment with 100µg isotype or anti-CD4 oropharyngeally administered or 500µg isotype or anti-CD4 intraperitoneally administered. *p<0.05, **p<0.01, ***p<0.005, ****p<0.0005 by two-way ANOVA with multiple comparisons. **D**.) ILC2 number was similar day 7 post-nV/aV in antibody treated groups.

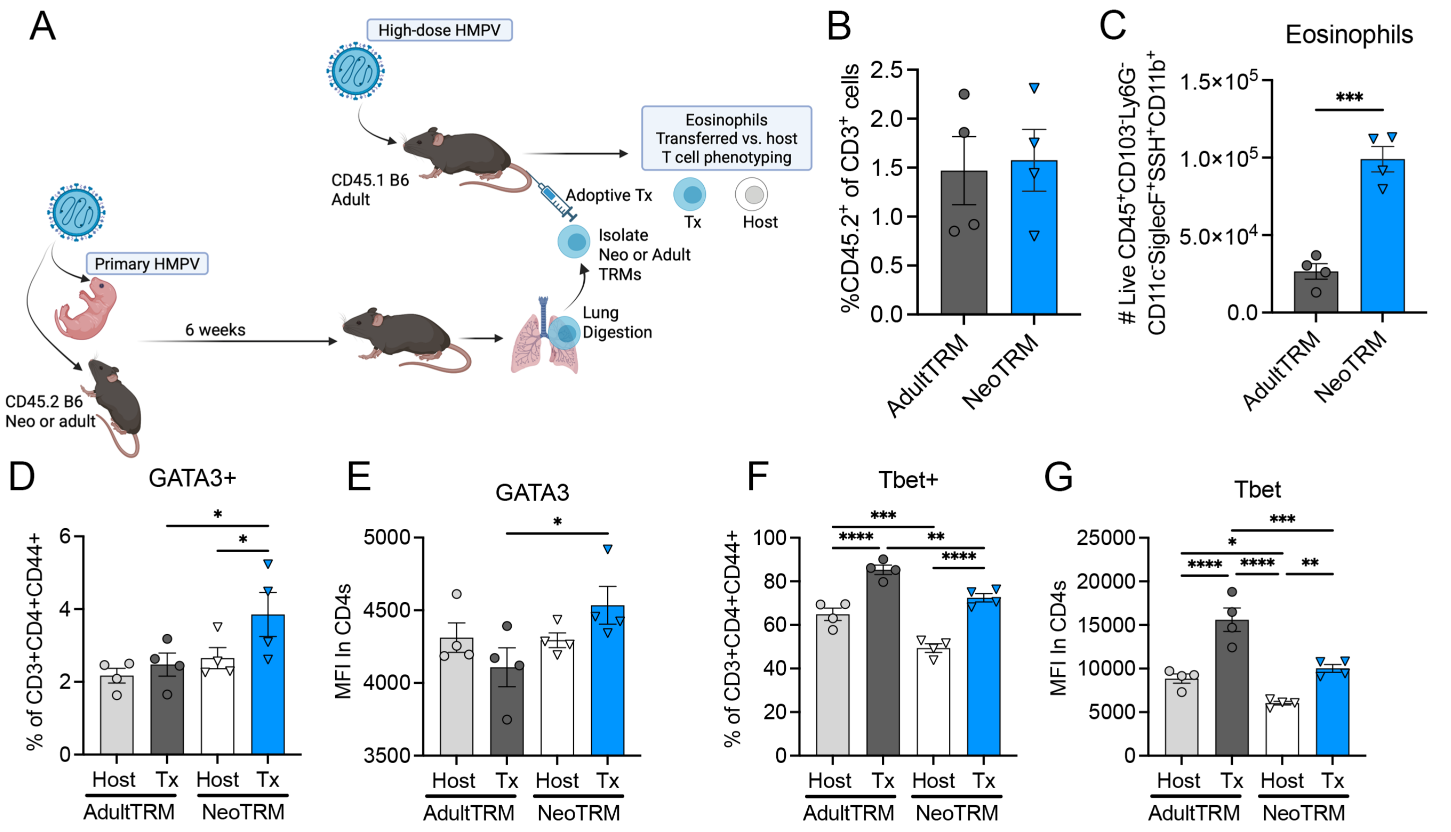

Supplemental Figure 8. Adoptive transfer of NeoTRMs maintains Th2 features. A.) Schematic of NeoTRM and AdultTRM generation, isolation, and adoptive transfer with concomitant HMPV infection. Created with Biorender.com. Mice were then euthanized at day 7 post-HMPV. **B**.) Quantification of CD45.2 donor T cells 7 days after HMPV. **C**.) Eosinophil quantification in mice receiving AdultTRM or NeoTRM. ***p<0.005 by student's t-test. **D-E**.) GATA3 MFI and frequency in donor and host $CD4^+$ T cells isolated from mice with transferred Adult or Neo TRMs. **F-G**.) Tbet MFI and frequency in donor and host $CD4^+$ T cells isolated from mice with transferred Adult or Neo TRMs. *p<0.05, ***p<0.005, ****p<0.0005 by one-way ANOVA with multiple comparisons.

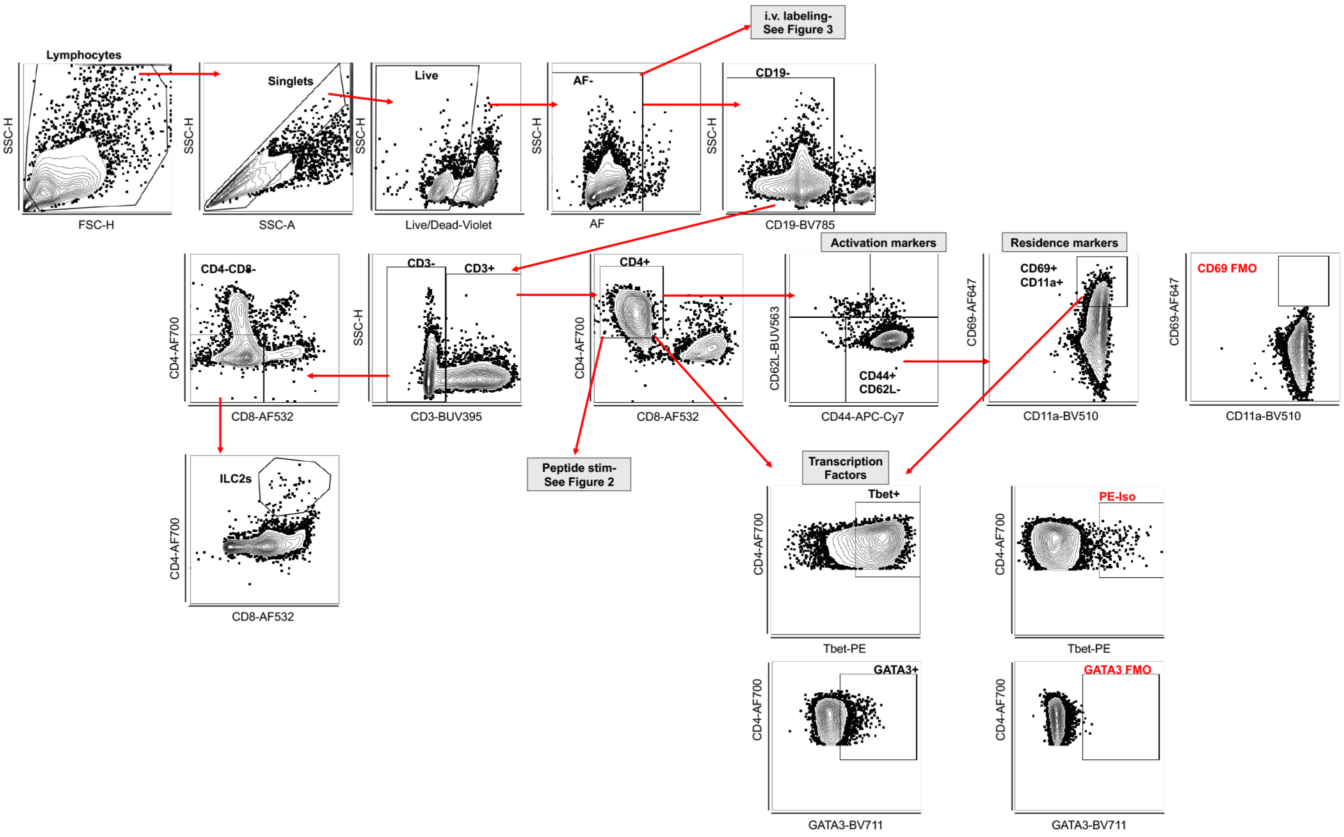

Supplemental Figure 9. Adaptive immune gating strategy. Pertinent FMOs or isotype control samples shown on right.

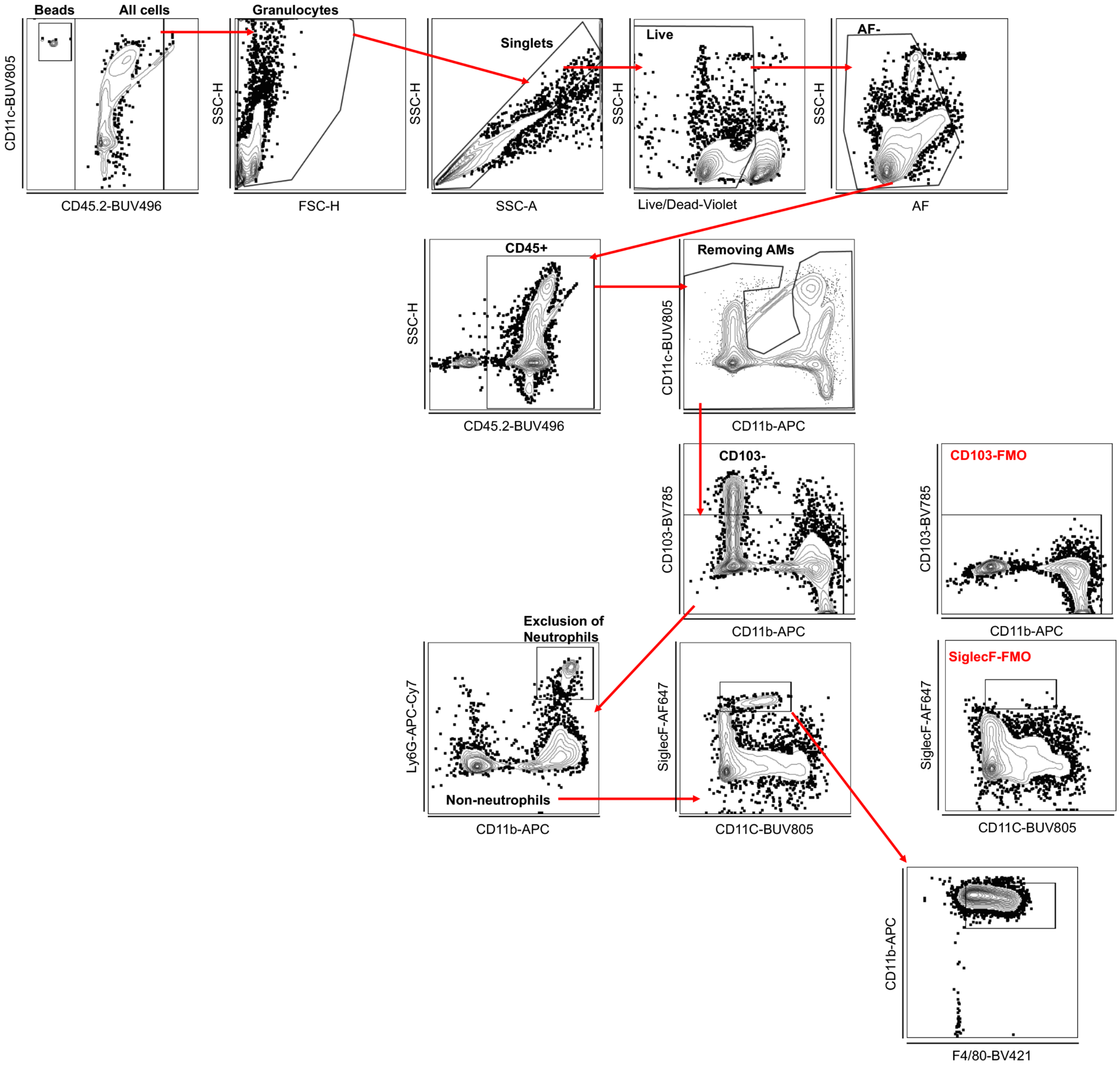

Supplemental Figure 10. **Innate immune gating strategy**. Pertinent FMOs shown on right.

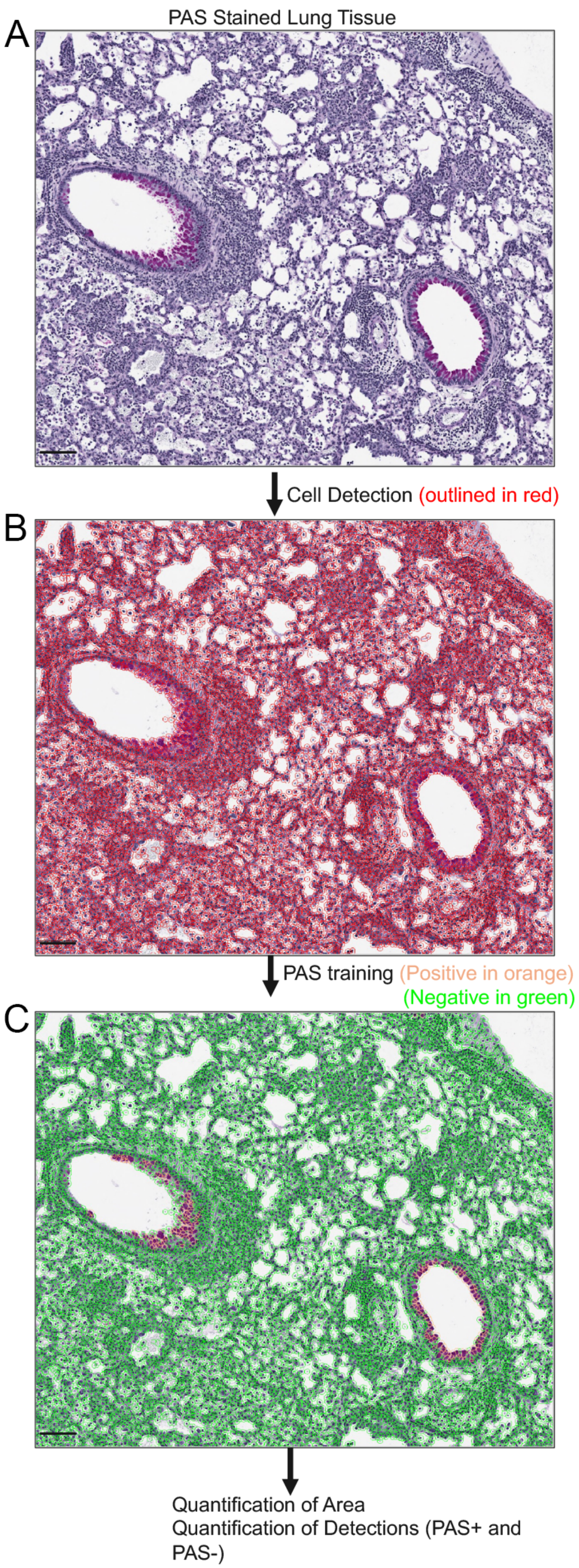

Supplemental Figure 11. **PAS quantification method**. **A**.) Representative PAS stained lung tissue. Scale bar= 100μm. **B**.) Cellular detection, with each individual cell outlined in red, providing a denominator of total cells. **C**.) Training on PAS+ and PAS- cells allows for separation of PAS+ (orange) or PAS- (green) cells, allowing for detection of PAS+ cells. Total lung area can also be quantified from lung tissue.
